# Feedback regulation by trehalose 6‐phosphate slows down starch mobilization below the rate that would exhaust starch reserves at dawn in Arabidopsis leaves

**DOI:** 10.1002/pld3.78

**Published:** 2018-08-13

**Authors:** Letícia dos Anjos, Prashant Kumar Pandey, Thiago Alexandre Moraes, Regina Feil, John E. Lunn, Mark Stitt

**Affiliations:** ^1^ Max Planck Institute of Molecular Plant Physiology Potsdam Golm Germany; ^2^ Universidade Federal do Ceará Fortaleza Brazil; ^3^ Present address: National Research Council Canada (NRC‐CNRC) 110 Gymnasium Place Saskatoon Saskatchewan S7N 0W9 Canada

**Keywords:** Arabidopsis, circadian clock, diel, starch, trehalose 6‐phosphate

## Abstract

Trehalose 6‐phosphate (Tre6P), a sucrose signaling metabolite, inhibits transitory starch breakdown in Arabidopsis (*Arabidopsis thaliana*) leaves and potentially links starch turnover to leaf sucrose status and demand from sink organs (Plant Physiology, 163, 2013, 1142). To investigate this relationship further, we compared diel patterns of starch turnover in ethanol‐inducible Tre6P synthase (iTPS) lines, which have high Tre6P and low sucrose after induction, with those in *sweet11;12* sucrose export mutants, which accumulate sucrose in their leaves and were predicted to have high Tre6P. Short‐term changes in irradiance were used to investigate whether the strength of inhibition by Tre6P depends on starch levels. *sweet11;12* mutants had twofold higher levels of Tre6P and restricted starch mobilization. The relationship between Tre6P and starch mobilization was recapitulated in *iTPS* lines, pointing to a dominant role for Tre6P in feedback regulation of starch mobilization. Tre6P restricted mobilization across a wide range of conditions. However, there was no correlation between the level of Tre6P and the absolute rate of starch mobilization. Rather, Tre6P depressed the rate of mobilization below that required to exhaust starch at dawn, leading to incomplete use of starch. It is discussed how Tre6P interacts with the clock to set the rate of starch mobilization.

## 1 INTRODUCTION

Plants use light energy to drive photosynthetic carbon (C) gain, metabolism, and growth, but at night depend on C reserves accumulated in previous light periods. In many species, including Arabidopsis, foliar starch is the major C reserve (Smith & Stitt, 2007). Diel regulation of starch turnover may depend on the conditions (Paul & Foyer, 2001). In source‐limited plants, C is in short supply and it is crucial to manage C reserves to insure rapid investment in growth while avoiding C starvation at night (Scialdone and Howard, 2015; Smith & Stitt, 2007; Stitt & Zeeman, 2012). In sink‐limited conditions, C regulation of metabolism and growth is relaxed (Baerenfaller et al., 2015; Sulpice et al., 2014) and starch often accumulates in leaves and other parts of the plant. This incomplete utilization of starch may be at least partly due to feedback inhibition of starch mobilization by the sucrose signal trehalose 6‐phosphate (Figueroa and Lunn, 2016; Lunn, Delorge, Figueroa, Van Dijck, & Stitt, 2014; Martins et al., 2013). The following experiments provide further evidence that Tre6P plays a key role in the feedback regulation of starch mobilization. In particular, we ask whether feedback inhibition by Tre6P is minimized to allow full use of starch in conditions where C is in short supply, but operates effectively when C is in excess.

When Arabidopsis plants grow in conditions where less C is available per 24 hr cycle, they accumulate a larger proportion of their fixed C to starch in the daytime and slow down mobilization of starch during the night, compared to plants growing with a large C supply. As a result, starch reserves are almost but not completely exhausted at dawn, irrespective of the overall availability of C. This pattern of diel starch turnover maximizes growth in low C conditions by insuring that almost all of the fixed C are invested in growth within a 24 hr cycle, while avoiding a deleterious period of C starvation at the end of the night (EN; Geiger and Servaites 1994; Ishihara, Obata, Sulpice, Fernie, & Stitt, 2015; Smith & Stitt, 2007; Stitt & Zeeman, 2012). Starch turnover shows this diel profile in many species including important crops (Chatterton & Silvius 1979, 1980, 1981; Cheng, Moore, & Seemann, 1998; Matt et al., 2001; Mullen & Koller, 1988; Silvius, Chatterton, & Kremer, 1979; reviewed in Smith & Stitt, 2007).

Arabidopsis maintains this pattern of diel starch turnover across a wide range of growth conditions including different photoperiods (Gibon, Pyl, Sulpice, Höhne, & Stitt, 2009; Sulpice et al., 2014), light intensities (Mengin et al., 2017), and night temperatures (Pilkington et al., 2015; Pyl et al., 2012). In addition, and crucially, sudden perturbations in growth conditions trigger a change in the rate of starch mobilization such that starch reserves last until the coming dawn. Examples include slowing down of mobilization after a sudden early dusk (Feike et al., 2016; Graf, Schlereth, Stitt, & Smith, 2010; Martins et al., 2013; Scialdone et al., 2013) or a single low light (LL) day (Feike et al., 2016; Pilkington et al., 2015), and speeding up of mobilization after a sudden late dusk (Scialdone et al., 2013) or an interruption of the night with a short interval of light (Scialdone et al., 2013). These rapid responses will be important in optimizing growth in a fluctuating environment.

These observations prompted the idea that starch mobilization is timed to the next dawn by the circadian clock (Graf et al., 2010; Scialdone et al., 2013). There are several lines of evidence for involvement of the clock. Wild‐type plants growing in a 17‐hr or a 28‐hr light‐dark cycle exhaust their starch at about 24 hr after the previous dawn, reflecting the innate 24‐hr periodicity of the wild‐type clock. The *lhy cca1* mutant exhausts its starch about 18–20 hr after the previous dawn, matching the shortened period in this mutant (Graf et al., 2010; Scialdone et al., 2013). Several models have been proposed to explain how the clock might regulate starch turnover (Dodd, Dalchau, Gardner, Baek, & Webb, 2014; Pokhilko, Flis, Sulpice, Stitt, & Ebenhöh, 2014; Scialdone and Howard, 2015; Scialdone et al., 2013; Seki et al., 2017). Some models propose that metabolic signals related to C availability modulate clock gene expression or clock period (Seki et al., 2017; Shin et al., 2017) or clock output pathways (Pokhilko et al., 2014). The arithmetic division model (Scialdone et al., 2013) proposes that a clock‐dependent mechanism measures time until the next dawn (*T*) and that this information is integrated with a measure of the starch content (*S*) to set the rate of starch mobilization (*R* = *S*/*T*). The robust pacing of starch mobilization to the coming dawn in the face of sudden perturbations (see above) is explained well by the molecular division model, but less easily by models that invoke changes in clock periodicity. However, it remains unclear which molecular outputs from the clock regulate the rate of starch mobilization, and how the amount of starch is measured. The biochemical mechanism that sets the rate of mobilization is also not known. There is some evidence that changes in the rate of starch mobilization may be linked with changes in phosphorylation of starch (Martins et al., 2013; Scialdone et al., 2013), and *early starvation1* (*esv1*) mutants were recently described in which premature exhaustion of starch is associated with altered granule structure and shape (Feike et al., 2016).

The widespread elevation of crop yield in Free Air CO_2_ Enhancement studies points to C supply often being at least partly limiting for plant growth in the field (Long, Ainsworth, Leakey, Nösberger, & Ort, 2006). In some conditions, like nutrient‐limiting conditions, C gain does not limit growth (Körner, 2006). Sink‐limited plants do not fully exhaust their starch (Grimmer, Bachfischer, & Komor, 1999; Hädrich et al., 2012; Lawlor & Mitchell, 1991; Pilkington et al., 2015), implying that feedback mechanisms related to low demand for C depress the rate of starch mobilization below that which would be allowed by the clock. In some cases, starch is exhausted in source leaves but reserves remain elsewhere in the plant (Czedik‐Eysenberg et al., 2016).

While it has been known for a long time that starch accumulates in sink‐limited plants, an underlying mechanism was only recently uncovered. Trehalose 6‐phosphate (Tre6P) has been proposed to act as a sucrose signal to regulate sucrose homeostasis (Figueroa and Lunn, 2016; Lunn et al., 2006, 2014; Yadav et al., 2014). Martins et al. (2013) used ethanol‐inducible TPS‐overexpressor *(iTPS*) lines to investigate whether Tre6P regulates the rate of mobilization of leaf starch. *iTPS* plants were grown in a 12‐hr photoperiod for 3 weeks and then induced at the end of the day (ED) to increase Tre6P levels in the following night. This led to slower starch mobilization and strongly decreased levels of maltose (Martins et al., 2013), an early intermediate in foliar leaf mobilization (Lu & Sharkey, 2006; Niittylä et al., 2004; Stitt & Zeeman, 2012), as well as a large decrease in the levels of sucrose and reducing sugars (Martins et al., 2013). These results indicated that Tre6P exerts feedback regulation on starch mobilization that Tre6P acts at an early step in the mobilization pathway and that high Tre6P is able to override any other signals that might be generated by changes in sugar levels. However, the molecular mechanism by which Tre6P inhibits starch mobilization remains unclear. Furthermore, the action and efficacy of Tre6P has only been analyzed in *iTPS* lines and in one condition in which (see Sulpice et al., 2014) the plants were slightly source limited. We also lack studies that document the full chain of events in which a decreased demand or rate of export of sucrose acts via an increase in Tre6P to slow down starch mobilization.

Export of sucrose in Arabidopsis involves its release to the apoplast by the sucrose effluxers *SWEET11* and *SWEET12* (Chen et al., 2012) followed by active uptake into the phloem complex by sucrose‐H^+^ cotransporters (Ayre, 2011; Lemoine et al., 2013; Riesmeier, Hirner, & Frommer, 1993). Mutants deficient in SWEET proteins show a reproducible but relatively small increase in leaf sucrose, compared to the drastic increases seen in mutants deficient in sucrose‐H^+^ cotransporters (Chen et al., 2012). They also bring the advantage that there is no accumulation of sucrose in the apoplast, which can trigger complex signaling and biotic resistance responses (Bolouri Moghaddam & Van den Ende, 2012; Herbers et al., 2000; Lemoine et al., 2013).

In the following experiments, we first asked whether the elevated levels of sucrose in leaves of *sweet* mutants lead to an increase in Tre6P and an inhibition of starch mobilization. We then present experiments with *sweet* mutants in conditions in which dusk starch levels were either low or high to learn whether the effectiveness of Tre6P depends on C status. In a parallel set of experiments with *iTPS* lines, we investigated the relationship between increased Tre6P levels and starch mobilization when sucrose and other sugars are falling (see above) rather than rising. The results strengthen the evidence that Tre6P plays a key role in the feedback regulation of starch mobilization. Rather surprisingly, they also reveal that there is no simple relationship between the Tre6P level and the rate of starch mobilization. Rather, Tre6P depresses the rate of mobilization below that required to exhaust starch at the coming dawn. This finding points to a close link between the mechanisms by which Tre6P and the clock act on starch mobilization.

## 2 MATERIALS AND METHODS

### 2.1 Plant material

Wild‐type *Arabidopsis thaliana* (L.) Heyhn. accession Columbia‐0 (Col‐0) was provided by in‐house collection (MPIMP, Golm). The Arabidopsis (Col‐0) ethanol‐inducible TPS‐overexpressor (*iTPS*) and empty‐vector control *AlcR* lines were the same as those described in (Martins et al., 2013).The Arabidopsis (Col‐0) *sweet11*,* sweet12,* and *sweet11,12* mutants and wild‐type segregant line (Chen et al., 2012) were kindly provided by Prof. Wolf Frommer (Heinrich Heine University, Düsseldorf, Germany).

### 2.2 Plant growth conditions and ethanol induction

Seeds were sown in soil mixed with vermiculite (1:1) in 10‐cm pots and placed in a phytotron under long‐day (LD) conditions, 16 hr (20°C) light/8 hr (4°C) dark, with an irradiance of 160 μmol m^−2^ s^−1^. After 1 week, pots were transferred directly to equinoctial conditions (12 hr light/12 hr dark) at constant 20°C, with an irradiance of 160 μmol m^−2^ s^−1^. At 2 weeks after sowing, seedlings were transplanted into 10‐cm pots (5 plants per pot). For the continuous light experiment, seeds were sown on soil in 10‐cm pots and placed directly in a phytotron under continuous light with a constant irradiance of 160 μmol m^−2^ s^−1^ and constant temperature of 20°C. At 2 weeks after sowing, plants were thinned out inside the phytotron, leaving five plants per pot.

Short‐term responses were evaluated at 21 days after sowing by submitting a batch of plants to different growth conditions. On the day of the experiment, some of the wild‐type Col‐0 and *sweet11;12* mutant plants was transferred at dawn to either a LD (15 hr light/9 hr dark, 160 μmol m^−2^ s^−1^, 20°C), or high light (HL; 12 hr/12 hr dark, 320 μmol m^−2^ s^−1^,20°C) or to combined LD and HL (LD + HL; 15 hr light/9 hr dark, 320 μmol m^−2^ s^−1^, 20°C). Similarly, on the day of the experiment, some of the empty‐vector control *AlcR* and *iTPS* plants were transferred at dawn to HL; 12 hr (320 μmol m^−2^ s^−1^, 20°C) or to LL (80 μmol m^−2^ s^−1^, 20°C) Plants were induced by spraying the mutant and the empty‐vector control *AlcR* 2 hr before dusk with 2% (v/v) ethanol.

For the continuous light experiment, empty‐vector control *AlcR* and *iTPS* plants were germinated, thinned to four plants per pot, and grown in continuous light at 20°C for 3 weeks. On the day of the experiment, plants were sprayed with 2% (v/v) ethanol and then darkened 2 hr later.

In all experiments, plants were harvested just before darkening and at various times during the night, as indicated for each individual experiment. Whole rosettes were excised under the ambient conditions and immediately quenched in liquid nitrogen. For sampling in the light, care was taken not to shade the leaves at any time before quenching. Plants from one or two pots (i.e., five or 10 plants) were pooled to form one sample, ground to a fine powder at −70°C using a robotized ball mill (Smith & Stitt, 2007), subaliquoted in a cryorobot, and stored at −80°C until use.

### 2.3 Extraction and measurement of metabolites

Starch, glucose, fructose, and sucrose were determined by enzymatic assays in ethanolic extracts of 20 mg frozen plant material as described by Cross et al. (2006). Assays were performed in 96‐well microplates using a Janus pipetting robot (Perkin Elmer, Wellesley, MA, USA; www.perkinelmer.com). Absorbances were determined using a Synergy, an ELx800 or an ELx808 microplate reader (Bio‐Tek, Bad Friedrichshall, Germany; www.bio-tek.com). For all the assays, two technical replicates were determined per biological replicate. Tre6P and ADP‐glucose (ADPGlc) were extracted in chloroform‐methanol and measured by high‐performance anion‐exchange chromatography coupled to tandem mass spectrometry (LC‐MS/MS) as described by Lunn et al. (2006) with modifications as in Figueroa et al. (2016). Tre6P was quantified using enzymatically calibrated standards and a [_2_H]Tre6P internal standard to correct for ion suppression and matrix effects (Lunn et al., 2006).

*Transcript analysis* was performed essentially as in Flis et al. (2015, 2016). mRNA was extracted from samples using an RNeasy Plant Mini kit (Qiagen; www.qiagen.com). cDNA was synthesized using a SuperScript III First‐strand Synthesis System Kit (Invitrogen; www.thermofisher.com). Quantitative real‐time PCR was performed using the SYBR Green PCR kit (Qiagen). Data were collected using SDS software (Applied Biosystems; www.appliedbiosystems.com). Concentration of target gene transcripts in the samples was calculated using standard curves generated for each sample, based on *C*_t_ values and spike‐in controls added to the samples before RNA extraction (Flis et al., 2015; Piques, Schulze, Gibon, Rohwer, & Stitt, 2009).

## 3 RESULTS

### 3.1 Kinetics of starch breakdown in the *sweet11;12* mutant

**Figure 1:**
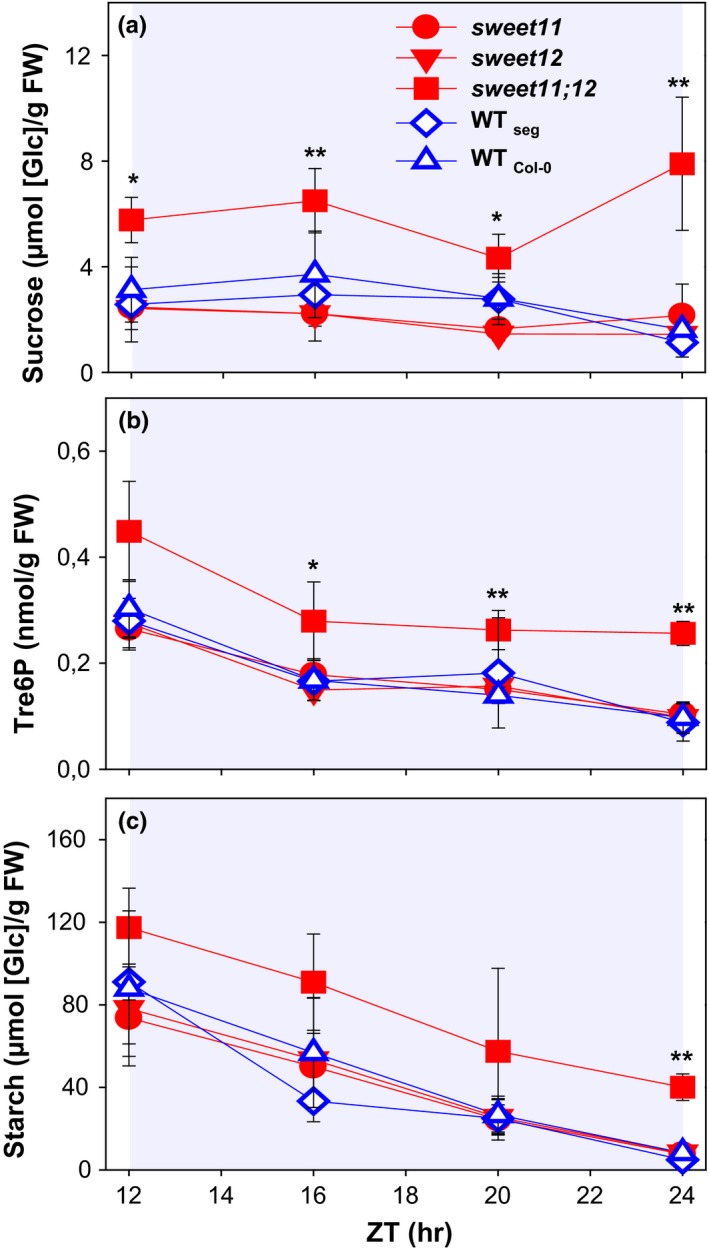
Night‐time metabolite levels in *Arabidopsis thaliana sweet* mutants in standard growth conditions. Arabidopsis *sweet11*,* sweet12,* and *sweet11;12* mutants were grown in standard conditions (12‐hr/12‐hr light/dark cycle, 21°C, 160 μmol m^−2^ s^−1^ PFD) along with a wild‐type segregant (WT_seg_ and wild‐type Col‐0, WT_Col0_) plants, and rosettes were harvested at various times during the night. (a) Sucrose, (b) Tre6P, (c) Starch. Values shown are mean ± *SD* (*n* = 3). Significant differences between *sweet11;12* and WT_C__ol0_, using Student's *t* test are indicated by asterisks: *p < 0.05,**p < 0.01, and ***p < 0.001. The original data are provided in Supporting Information Table S1; glucose and fructose are shown in Supporting Information Figure S1. ZT, zeitgeber time (hours after dawn)

Wild‐type Col‐0, the *sweet11* and *sweet12* mutants, and the *sweet11;12* double mutant were grown in a 12‐hr photoperiod with 160 μmol m^−2^ s^−1^ photon flux density (PFD) at rosette level for 30 days and then sampled at dusk and 4‐hr intervals during the night (Figure 1, original data provided in Supporting Information Table S1). Two Col‐0 wild‐type lines were included; one from standard seed stock, and one that segregated from the cross between *sweet11* and *sweet12* (Col‐0_(seg)_).

In the wild‐type lines, sucrose levels were fairly constant throughout the night. The levels (about 2 μmol/g FW) resembled those seen previously in our standard growth conditions (12‐hr photoperiod, 160 μmol m^−2^ s^−1^ m 20°C; Annunziata et al., 2017; Flis et al., 2016; Mengin et al., 2017; Pal et al., 2013; Sulpice et al., 2014). Sucrose was not significantly changed in the single *sweet* mutants, but was elevated two‐ to threefold in the double mutant (Figure 1a; see also Chen et al., 2012). Glucose and fructose (Supporting Information Figure S1) were very low throughout the night in the wild‐types, not significantly increased in the single mutants, and strongly increased in *sweet11;12*. Tre6P (Figure 1b) was unaltered in the single mutants, and 80%–150% increased in *sweet11;12* compared to wild‐type lines. In wild‐type lines, starch decreased during the night in a linear manner and was almost completely exhausted at dawn (Figure 1c), as seen in many previous studies (Annunziata et al., 2017; Flis et al., 2016; Gibon et al., 2004; Graf et al., 2010; Mengin et al., 2017; Pal et al., 2013; Scialdone et al., 2013). Starch mobilization in the single mutants resembled Col‐0. At dusk, *sweet11;12* contained about 30% more starch than the wild‐types or the single mutants. Unexpectedly, *sweet11;12* broke its starch down at almost the same rate as the other three genotypes. As a result of the high dusk starch content, *sweet11;12* had a large starch excess at dawn. Of the starch at dusk, <10% remained at dawn in wild‐type lines and the single *sweet* mutants, but about 37% in *sweet11;12*.

### 3.2 Effect of increased night‐time sucrose and Tre6P levels on starch mobilization

**Figure 2:**
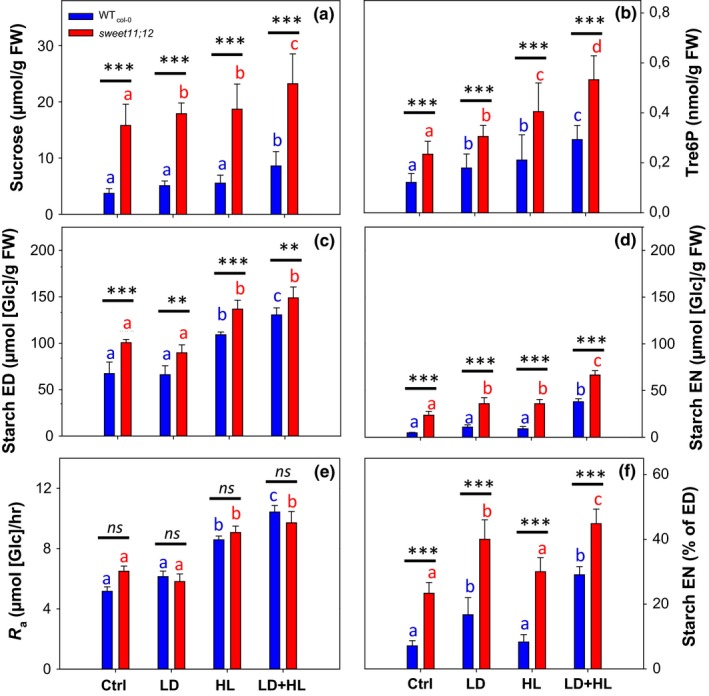
Effect of short‐term treatments to alter dusk starch content on night‐time levels of Tre6P and sucrose, starch content at dusk and dawn and starch mobilization in wild‐type Col‐0 and the *sweet11;12* mutant. Wild‐type Col‐0 plants (WT) and *sweet11;12* double mutants were grown in standard conditions (12‐hr/12‐hr light/dark cycle, 21°C, 160 μmol m^−2^ s^−1^ PFD). Three weeks after germination, batches of plants were left in standard conditions (Ctrl) or were shifted for a single day either to a long‐day condition (LD, 15‐hr photoperiod, 21°C, 160 μmol m^−2^ s^−1^ PFD), or to high‐light condition (HL, 12‐hr photoperiod, 21°C, 320 μmol m^−2^ s^−1^ PFD), or a combined LD and high‐light condition (LD + HL). Samples were collected at the end of the light period (ED) after the shift, and throughout the following dark period at 3‐hr intervals, up to the end of the night (EN) for metabolite analysis. (a) Night‐time sucrose and (b) night‐time Tre6P levels, averaged from ZT15 to ZT24, see Supporting Information Figure S3 for values at each time; (c) starch content at ED; (d) starch content at EN; (e) absolute rate of starch mobilization (*R*_a_), estimated from the slope of the linear regression of starch contents measured along the night from dusk to dawn (see Figure 3); (f) starch content at EN expressed as a percentage of starch content at ED. Values are mean ± *SD* (*n* = 5). Asterisks indicate statistically significant genotype‐dependent differences in a given treatment: *p < 0.05, **p < 0.01, and ***p < 0.001. Letters indicate significant treatment‐dependent differences in a given genotype; blue letters indicate treatment differences within WT_C__ol‐0,_ and red letters indicate differences within *sweet11;12* double mutant (one‐way ANOVA, Holm‐Sidak post hoc pairwise multiple comparison testing, *p* < 0.01). The original data are provided in Supporting Information Table S2, and the temporal changes of Tre6P, sucrose, and other selected metabolites are shown in Supporting Information Figure S2. A replicate experiment for the Crtl, LD, and HL treatments is shown in Supporting Information Figures S3 and S4. ZT, zeitgeber time (hours after dawn)

**Figure 3:**
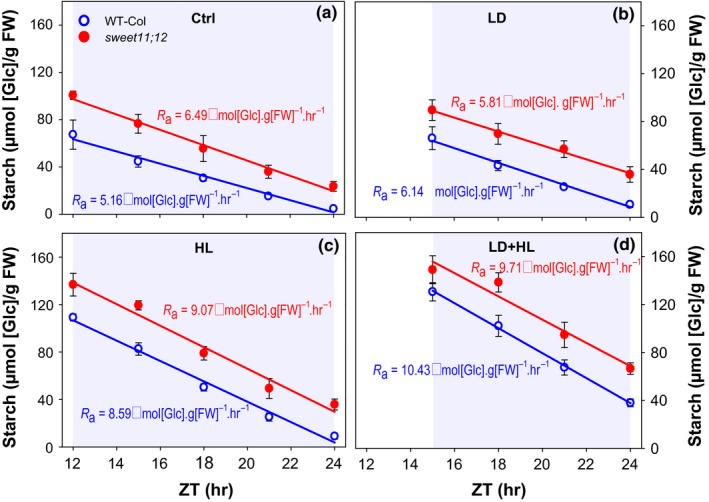
Effect of short‐term treatments to alter dusk starch content on the temporal kinetics of starch mobilization in wild‐type Col‐0 and the *sweet11;12 double* mutant. This is a further analysis of data from the experiment shown in Figure 2. Wild‐type Col‐0 plants (WT) and *sweet11;12* double mutants were grown in standard conditions (12‐hr/12‐hr light/dark cycle, 21°C, 160 μmol m^−2^ s^−1^ PFD) for 3 weeks after germination. On the day of the experiment, plants were either left in the standard growth condition (a) or shifted to a new condition for a single day to increase starch content at dusk: (b) high‐light (HL, 12‐hr photoperiod, 21°C, 320 μmol m^−2^ s^−1^ PFD); (c) long‐day (LD, 15‐hr photoperiod, 21°C, 160 μmol m^−2^ s^−1^ PFD); or (d) long‐day and high‐light (LD + HL). Rosettes were harvested at dusk and at 3‐hr intervals during the night to measure starch content. Values are means ± *SD* (*n* = 5). Lines show the linear regressions of starch content from which the absolute rate of starch mobilization (*R*_a_) was calculated (numbers given in panel). ZT, zeitgeber time (hours after dawn)

We were curious whether the generally elevated levels of starch and unaltered rate of starch mobilization in *sweet11;12* were a consequence of the conditions used in the experiment of Figure 1, or a robust phenotype of this mutant. Wild‐type plants and *sweet11;12* were grown in standard conditions (12‐hr photoperiod, 21°C, PFD of 160 μmol m^−2^ s^−1^ at rosette level) for 21 days, and batches were then either left in standard conditions (control; Ctrl) or shifted for a single day to a 15‐hr photoperiod (LD) with unaltered light intensity (160 μmol m^−2^ s^−1^), or to high‐light (HL, 320 μmol m^−2^ s^−1^ PFD, which is close to Arabidopsis light‐saturation point, see Pilkington et al., 2015) in a 12‐hr photoperiod, or to a combination of LD and HL (LD + HL). These treatments were chosen to generate different starch contents at dusk. Starch, sucrose, and Tre6P contents were investigated at the ED, at 3‐hr intervals during the night, and at the EN (Figures 2 and 3, Supporting Information Figure S2; original data provided in Supporting Information Table S2).

In wild‐type plants, the average night‐time levels of sucrose (Figure 2a) and Tre6P (Figure 2b) rose progressively between the Ctrl, LD, HL, and LD + HL treatments, with the increase being significant in LD + HL for sucrose and in all treatments for Tre6P. In all treatments, *sweet11;12* had higher average night‐time sucrose and Tre6P levels than wild‐type plants (*p* < 0.001 in all cases). Sucrose levels were fourfold higher in *sweet11;12* than wild‐types in Ctrl, and about threefold higher in the LD, HL, or LD + HL treatments. Average night‐time Tre6P levels in *sweet11;12* were about twofold higher than wild‐type in all treatments. In wild‐type plants, starch content at dusk (Figure 2c) was unaltered in LD and rose significantly in HL and LD + HL, compared to Ctrl. The lack of response of dusk starch in LD is because starch mobilization in the light accelerates with the duration of the light period (Fernandez et al., 2017). Starch in wild‐type plants at dawn (Figure 1d) was very low in Ctrl, marginally higher in LD and HL and significantly higher in LD + HL. Starch at dawn was significantly higher in *sweet11;12* than the wild‐type in all treatments. Relative to the wild‐type value, the increase in *sweet11;12* was relatively small at dusk (14%–50%) and larger at dawn (76%–244%), with the relative increase being larger in Ctrl than in HL, LD, or LD + HL.

The first dedicated and regulated reaction in starch synthesis is catalyzed by ADPGlc pyrophosphorylase, leading to formation of ADPGlc. The higher rates of starch accumulation in HL and HL + LD were accompanied by a twofold increase in the dusk level of ADPGlc (Supporting Information Figure S2). The increase in Tre6P in *sweet11;12* did not lead to an increase in dusk levels of ADPGlc. This is consistent with the similar rates of starch accumulation between dawn and dusk in *sweet11;12* and wild‐type plants. It is also consistent with the lack of effect of elevated Tre6P on ADPGlc levels and starch accumulation previously reported by Figueroa et al. (2017). ADPGlc levels were negligible in the dark in wild‐type plants. They were also negligible in the *sweet11;12* mutant, despite its high sucrose levels during the night.

Figure 3 shows the kinetics of starch mobilization during the night. Both wild‐type Col‐0 and *sweet11;12* degraded their starch in a near‐linear manner during the night (Figure 3). The slope of the linear regression on starch contents at ZT12, ZT16, ZT20, and ZT24 (ZT, zeitgeber time, i.e., hours since previous dawn) was used to estimate the absolute rate of starch mobilization (*R*_a_; Figure 2e). Comparing across treatments, wild‐type plants mobilized their starch slightly faster after the LD and considerable faster after the HL and LD + HL treatments than in the control (5.16, 6.14, 8.59, and 9.71 μmol [Glc] g^−1^ FW hr^−1^ in Crtl, LD, HL, and LD + HL, respectively). This increase in HL and LD + HL reflects the increased dusk starch content. Comparing across treatments, a higher dusk starch content led to a faster starch mobilization in *sweet11;12* (6.49, 5.81, 9.97, and 10.43 μmol [Glc] g^−1^ FW hr^−1^ in Crtl, LD, HL, and LD + HL, respectively. The key finding, however, is that in a given condition, the absolute rate of starch mobilization was not substantially or consistently slower in *sweet11;12* than in wild‐type plants. Comparing *sweet11;12* to wild‐type, mobilization was slightly faster in Ctrl (6.5 compared to 5.16 μmol [Glc] g^−1^ FW hr^−1^, *p* = 0.05), marginally faster in HL and marginally slower in LD and HL + LD, withir none of differences being significant (*p* = 0.18, 0.65 and 0.34). Nevertheless, due to the differing starch content at dusk, *sweet11;12* plants did not completely degrade their starch reserves in any of the treatments whereas wild‐type plants remobilized almost all their reserves by dawn in all treatments except LD + HL. The starch content at the EN, expressed as a percentage of the dusk starch content, was about twofold higher in *sweet11;12* (28% in Ctrl rising to 40%–43% in LD, HL, and LD + HL) than in wild‐type plants (about 10% in Ctrl, rising to 20% in LD and HL, and 28% in LD + HL; Figure 2f). Thus, the increase in sucrose and Tre6P in *sweet11;12* did not strongly or consistently decrease the absolute rate of starch mobilization in the experiment of Figures 2 and 3; rather, it decreased the proportion of the starch that is degraded during the night.

In a further experiment with control, LD and HL treatments (Supporting Information Figure S3) in any given treatment sucrose was again about threefold (Supporting Information Figure S2A) and Tre6P about twofold (Supporting Information Figure S2B) higher in *sweet11;12* than Col‐0. Compared to the experiment of Figures 2 and 3, wild‐type plants contained more starch at dusk (Supporting Information Figure S3D). This may reflect small differences in the growth conditions affecting starch turnover; a 12‐hr photoperiod with this irradiance and fertilization regime is close to the point at which growth of wild‐type Col‐0 switches from being source‐ to sink‐limited (Sulpice et al., 2014). Compared to wild‐type plants, starch levels in *sweet11;12* showed a trend to slightly higher levels at dusk (Supporting Information Figure S3C, significant in control conditions) and a large increase at dawn (Supporting Information Figure S3D, significant in all conditions). Compared to wild‐type plants, *R*_a_ was slightly higher in *sweet11;12* in Ctrl (6.03 compared to 6.82 μmol [Glc] g^−1^ FW hr^−1,^ respectively, *p* = 0.074) and lower in LD (8.77 compared to 6.95 μmol [Glc] g^−1^ FW hr^−1^) and HL (9.03 compared to 6.56) μmol [Glc] g^−1^ FW hr^−1^; Supporting Information Figure S4A–C), with none of these differences being significant (Supporting Information Figure S3E). As in the experiment of Figures 2 and 3, compared to Col‐0, *sweet11;12* showed a large and highly significant twofold increase in the percentage of the dusk starch content that remained at dawn (Supporting Information Figure S3F). Thus, in this third independent experiment, an increase in sucrose and Tre6P again did not consistently slow starch mobilization but instead led to a decrease in the percentage of starch that was mobilized during the night.

### 3.3 Effect of induced changes in Tre6P levels on starch mobilization rate in carbon‐limiting and carbon‐replete conditions

To further investigate whether the incomplete mobilization of starch in *sweet11;12* was associated with changes in Tre6P, we investigated starch mobilization in an ethanol‐inducible TPS‐overexpressor (*iTPS*) line (Figueroa et al., 2016; Martins et al., 2013). In this line, Tre6P levels can be inducibly increased within 2 hr in the absence of any increase in sucrose; indeed, the induced increase of Tre6P leads to a decrease of sucrose and reducing sugars (see Introduction, also below for more data). We investigated the response of starch mobilization to an increase in Tre6P in conditions where the plants were C‐limited and the plants were C‐replete.

*iTPS* plants were grown in parallel with the empty‐vector control line, *AlcR,* in standard conditions for 4 weeks. One batch was then subjected to a single HL (320 μmol photons m^−2^ s^−1^) and another batch to a single LL (80 μmol photons m^−2^ s^−1^; not much above the light‐compensation point of 30 μmol m^−2^ s^−1^) light period. All plants were sprayed with 2% (v/v) ethanol 2 hrs before dusk to insure the inducible mutants would enter the night with increased Tre6P levels. Rosettes were harvested in the light just before the onset of darkness (ED) and at 2‐hr intervals for the first 10 hr of the night to quantify starch, sugars, Tre6P, and other metabolites.

**Figure 4:**
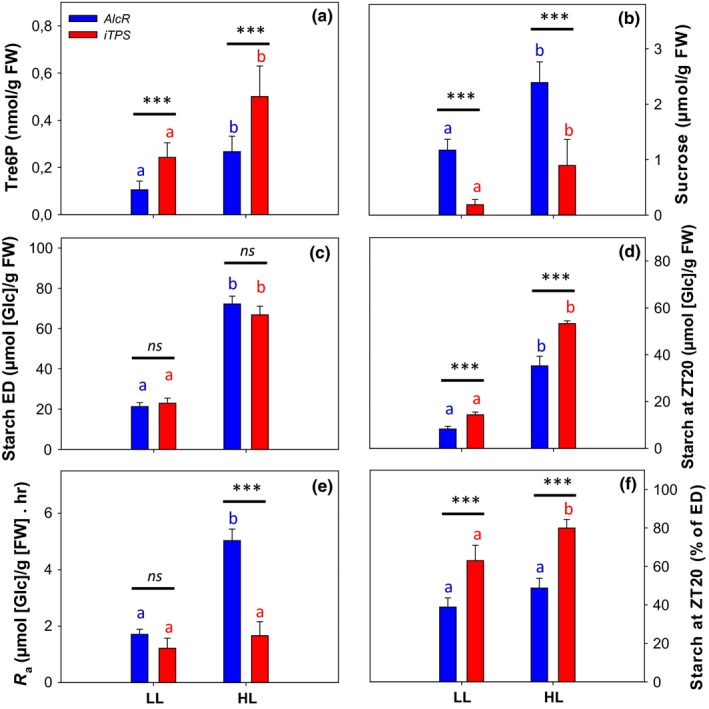
Effect of induced *TPS* overexpression on night‐time metabolite levels after short‐term perturbations to generate high or low starch content at dusk. The empty‐vector control *AlcR* and *iTPS* plants were grown under standard conditions (12‐hr/12‐hr light/dark cycle, 21°C, 160 μmol m^−2^ s^−1^ PFD). Three weeks after germination, batches of plants were shifted for a single day either to low light (LL, 12‐hr photoperiod, 21°C, 80 μmol m^−2^ s^−1^ PFD), or to high light (HL, 12‐hr photoperiod, 21°C, 320 μmol m^−2^ s^−1^ PFD). Plants were induced 2 hr before dusk by spraying with 2% (v/v) ethanol. Samples were collected at the end of the day (ED) after the shift, and throughout the following dark period at 2‐hr intervals, up to ZT20. (a) Night‐time sucrose and (b) night‐time Tre6P levels, averaged from ZT14 to ZT20, see Figure 5 for values at each time; (c) starch content at ED; (d) absolute rate of starch mobilization (*R*_a_), estimated from the slope of the linear regression on starch content between ZT12 and ZT20 (see Figure 6); (e) starch content at ZT20; (f) starch content at ZT20 expressed as a percentage of the starch content at ED (ZT12). Values are means ± *SD* (*n* = 5). Asterisks indicate statistically significant genotype‐dependent differences in a given treatment: *p < 0.05, **p < 0.01, and ***p < 0.001. Letters indicate significant treatment‐dependent differences in a given genotype: blue letters indicate treatment differences within WT_C__ol‐0,_ and red letters indicate differences within *sweet11;12* double mutant (one‐way ANOVA, Holm‐Sidak post hoc pairwise multiple comparison testing, *p* < 0.01). ZT, zeitgeber time (hours after dawn)

**Figure 5:**
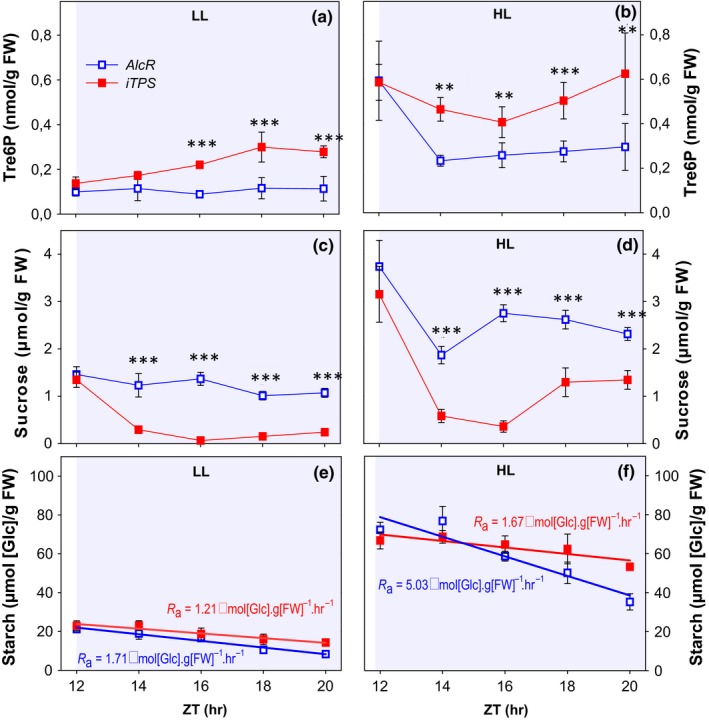
Effect of induced TPS overexpression on the temporal kinetics of Tre6P and sucrose levels and night‐time starch mobilization after short‐term perturbations to generate high or low starch content at dusk. This figure shows data from the same experiment as Figure 4. After one photoperiod in low light (LL) or high light (HL), empty‐vector control *AlcR* and the inducible TPS‐overexpressor line (*iTPS*) plants were induced 2 hr before dusk by spraying with 2% (v/v) ethanol. (a, b) Tre6P, (c, d) sucrose, and starch (d, e) in the LL treatment (a, c) or the HL treatment (b, d). Values are means ± *SD* (*n* = 5). In panels (e, f), lines represent the linear regressions of starch content from which the absolute rate of starch mobilization (*R*_a_) was calculated (numbers shown in panel). Significant differences between *AlcR* and *iTPS*, using Student's *t* test, are indicated by asterisks: *p < 0.05,**p < 0.01, and ***p < 0.001. ZT, zeitgeber time (hours after dawn)

Figure 4 shows the average Tre6P (Figure 4a) and sucrose (Figure 4b) levels during the night, starch content at ZT12 (ED) and ZT20 (Figure 4c, d) as well as the estimated average rate of starch mobilization (Figure 4e) and the starch content at ZT20 as a percentage of that at dusk (Figure 4f). Time kinetics are also provided for the changes of Tre6P (Figure 5a, b), sucrose (Figure 5c, d), and starch (Figure 5e, f). The original data are provided in Supporting Information Table S3.

Night‐time Tre6P levels were about twofold higher in *iTPS* than control *AlcR* plants (Figure 4a, b). This increase was highly significant (*p* < 0.001) when averaged over all times between ZT14 and ZT20 both in the LL and the HL treatment. Time resolved plots revealed that in the LL treatment (Figure 5a) Tre6P was low at dusk and remained low during the night in the control *AlcR* plants, whereas it rose steadily during the night in the induced *iTPS* line. In the HL treatment (Figure 5b), Tre6P was high at dusk and fell strongly in the first 4 hr of the night in the *AlcR* control, whereas it declined only slightly at 14 and 16 hr and then rose in *iTPS*. This resembles the response previously seen in plants grown in standard conditions and induced in the middle of the day or just before dusk (Martins et al., 2013).

Induction of *TPS* led to sucrose levels falling to under 20% and about 40% of the *AlcR* levels in the LL and HL treatment, respectively (Figure 4b). This decrease was significant within the first 2 hr of the night (Figure 5c, d). A large decrease in sucrose in the night after induction of *TPS* was seen previously (Martins et al., 2013) and attributed to an inhibition of starch mobilization by Tre6P. The decrease in sucrose was accompanied by a decrease of glucose and fructose (see Supporting Information Table S3).

Starch at dusk was nearly threefold higher in the HL than the LL treatment (Figure 4c). Starch was degraded in a near‐linear manner after the HL and LL treatments both in *AlcR* and *iTPS* (Figure 5e, f). *AlcR* plants mobilized less of their starch after the HL treatment than the LL treatment, with 49% and 39% of the dusk content remaining at ZT20. Extrapolation indicated that after the HL and LL treatments about 27% and 8% of their starch would remain at dawn. The response of an induced increase in Tre6P differed between the HL and LL treatments. Compared to the *AlcR* control, starch mobilization in *iTPS* was weakly but non‐significantly inhibited after LL, and significantly decreased after HL (to about 70% and 30% of the control rate, respectively, Figure 4e). The starch excess phenotype was prominent in *iTPS* in LL and very strong in HL with over 60% and 80%, respectively, of the starch remaining at ZT20 (Figure 4f). Extrapolation indicated that about 50% and 75% of the dusk starch would have remained at the next dawn. For comparison, in the study of Martins et al. (2013) using *iTPS* plants growing in similar conditions to those of Figure 4 but left in growth irradiance on the day on which *TPS* was induced, *iTPS* showed an approximately twofold increase in Tre6P compared to control plants after induction, and the starch content at dawn was about 60% of the starch content at dusk.

### 3.4 Integration of the response to an increase of Tre6P in the *sweet11;12* mutant and induced *iTPS* lines

**Figure 6:**
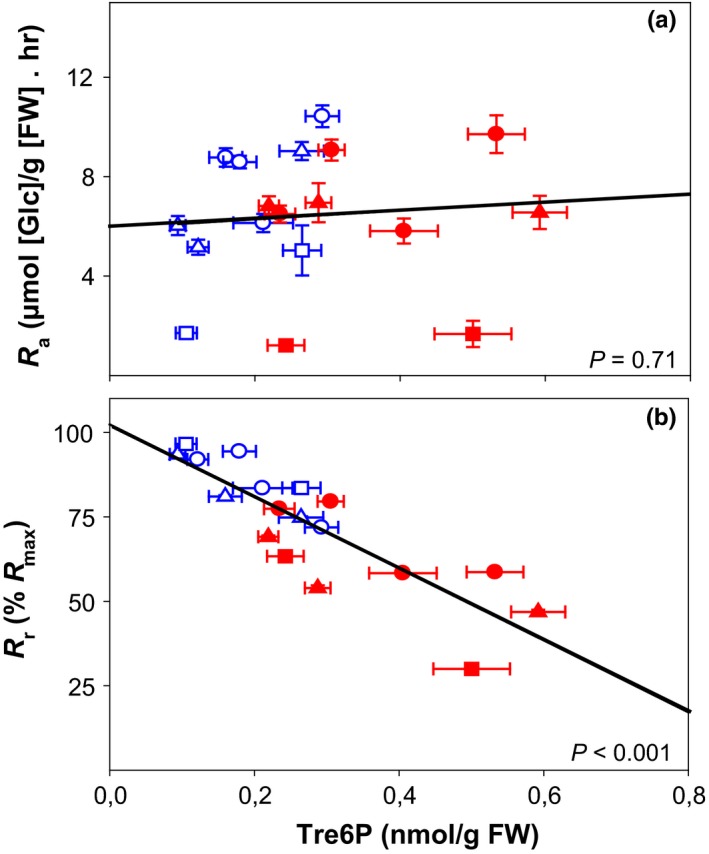
Correlation of average night‐time Tre6P contents and starch mobilization in Arabidopsis rosettes. The plots show the relation between Tre6P levels and (a) the absolute starch mobilization rate (*R*_a_), and (b) the relative starch mobilization rate (*R*_r_) in wild‐type Col‐0, *sweet11;12* double mutant, *AlcR* empty‐vector control and induced TPS‐overexpressor (*iTPS*) line grown under standard conditions and subjected for the preceding light period to different light intensities or to different photoperiods (for details see legends of Figures 2 and 4). Values for Tre6P are the average values between ZT15 and ZT24 for experiments with wild‐type Col‐0 and *sweet11;12* mutant (see Figure 2b, Supporting Information Figure S3B) and between ZT14 and ZT20 for the experiment with *AlcR* and *iTPS* (Figure 4b). Values for *R*_a_ are derived from Figure 3 and Supporting Information Figure S4 for wild‐type Col‐0 and *sweet11;12*, and from Figure 6 for *AlcR* and *iTPS*. *R*_r_ is calculated as *R*_a_/*R*_max_, where *R*_max_ is the rate of starch mobilization that would exhaust starch at EN. *R*_r_ is also geometrically equivalent to the proportion of starch at ED that would remain at EN. The underlying calculations are provided in Supporting Information Table S4

We next evaluated the impact of night‐time Tre6P level on starch mobilization in a combined data set, which contained the experiments with *sweet11;12* and the experiments with *iTPS*. There was no correlation between night‐time levels of Tre6P and the absolute rate of starch breakdown, *R*_a_ (Figure 6a).

Irrespective of whether we used *sweet11;12* or *iTPS*, an increase in Tre6P was associated with a higher starch content toward the EN. This indicated that Tre6P might depress the rate of starch mobilization relative to the amount of starch available at dusk. We calculated, for each experiment and reatment, the maximum rate of starch mobilization (*R*_max_) that could be supported by the dusk starch content under the provisos that breakdown is linear and that starch is not exhausted before dawn. *R*_max_ is equivalent to the slope of a linear regression through the measured starch content at ED and a hypothetical null starch content at EN (ZT24). We then estimated the extent to which starch mobilization was depressed relative to *R*_max_ (*R*_r_ = *R*_a_/*R*_max_) and compared *R*_r_ with the average night‐time Tre6P level. As shown in Figure 6b, there was a strong and highly significant negative correlation between Tre6P and *R*_r_ (*R* ^2^ = 0.72, *p* < 0.001). This strong relationship was found even though the starch content at dusk and absolute rate of starch mobilization varied considerably between the various treatments. Importantly, a similar relationship was found for *sweet11;12* and *iTPS*, even though the former had elevated sucrose and the latter had lower sucrose than control plants. This consistent match across genotypes strongly indicates that the negative relationship in Figure 6b is driven by Tre6P.

### 3.5 Impact of Tre6P on starch mobilization when the clock is desynchronized

The arithmetic division model (Scialdone et al., 2013) provides a simple conceptual framework to understand how starch breakdown is paced to dawn in a wide range of conditions including sudden perturbations (see Introduction). In this model, a clock‐dependent mechanism measures time remaining until the next dawn (‘*T*’). This information is integrated with a measure of the starch content (‘*S*’) to set the rate of starch mobilization (‘*R*’ = ’*S*’/’*T*’). The term *R*_max_ in the calculations underlying Figure 6b is analogous to term ‘*R*’ in the arithmetic division model. The negative correlation in Figure 6b is consistent with Tre6P depressing the rate of starch mobilization below that set by the clock.

We therefore asked whether the inhibition of starch mobilization by Tre6P depends on operation of the clock. To do this, we grew the *AlcR* and *iTPS* lines in continuous light and continuous temperature from germination onwards for 3 weeks and then turned the light off. Plants were induced with ethanol 2 hr prior to the onset of darkness. Rosettes were harvested at 4‐hr intervals in the 24 hr prior to the onset of the darkness, and at 2‐hr intervals for the first 12 hr after darkening.

We first checked whether the clock was desynchronized. To do this, we monitored transcript abundance of ten core clock genes (*LHY, CCA1*,* PRR9*,* PRR7*,* PRR5*,* GIGANTEA* (*GI*)*, TIMING OF CAB EXPRESSION 1* (*TOC1*), *EARLY FLOWERING3* (*ELF3*), *ELF4*, and *LUX ARRHYTHMO* (*LUX*)) as well as two output genes (*PHYTOCHROME‐INTERACTING FACTOR 4* (*PIF4)* and *PIF5*) in *AlcR* and *iTPS* lines in the last 24 hr of continuous light and the first 12 hr after darkening (Supporting Information Figure S5). The results are compared with oscillations of the same transcripts in 21‐day‐old wild‐type Col‐0 growing in a 12‐hr photoperiod (Flis et al., 2016). The latter time series is plotted such that dusk coincides with the time at which the *AlcR* and *iTPS* plants were darkened. The strong oscillations of clock, *PIF4,* and *PIF5* transcripts in a light‐dark cycle were abolished in continuous light, both in *AlcR* and in *iTPS*. In the first 12 hr after darkening, there was a small decrease of *PRR9*,* GI,* and *LUX* transcripts and a slow rise of *LHY*,* CCA1*,* PIF4,* and *PIF5* transcripts, but these changes were very small compared to the oscillations in plants grown in a light‐dark cycle.

**Figure 7:**
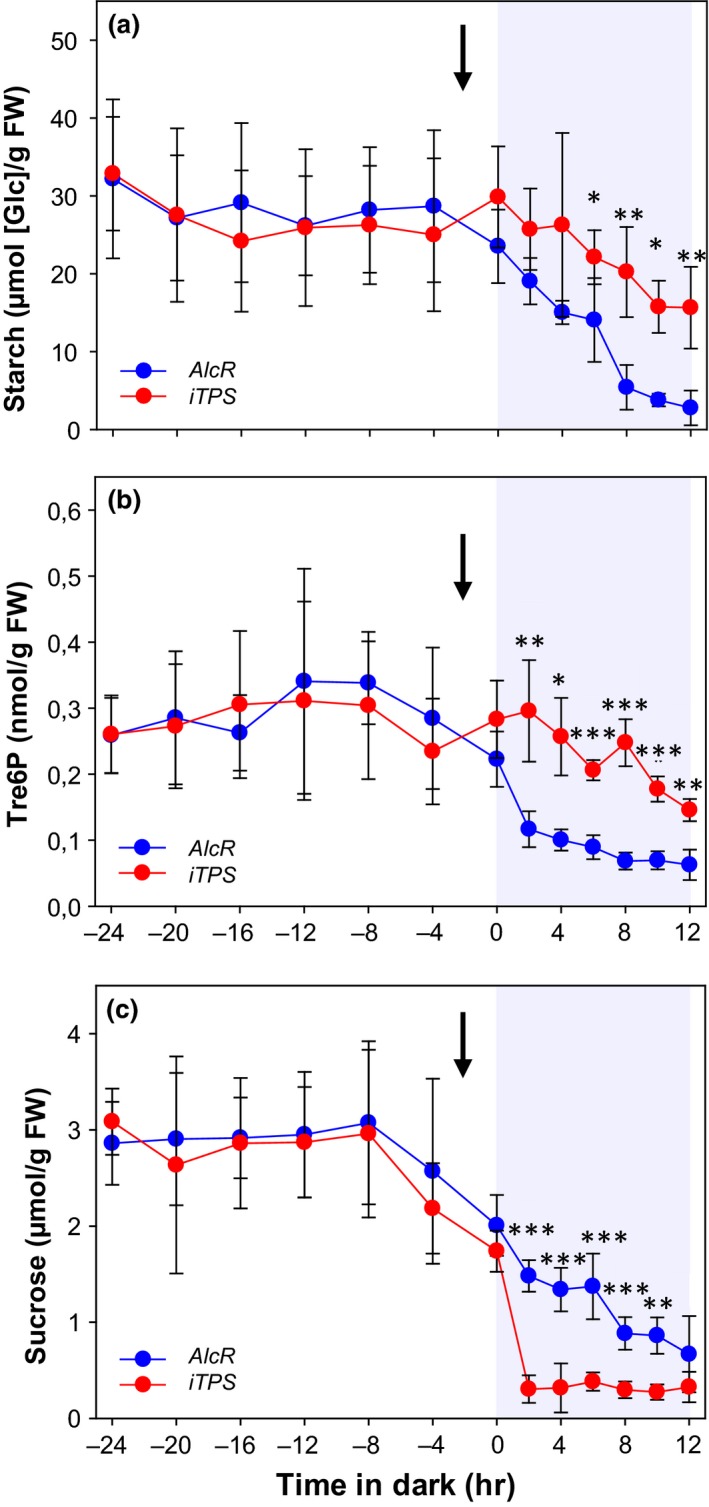
Effect of induced TPS overexpression on starch mobilization after darkening plants that were previously germinated and grown in continuous light. The empty‐vector control *AlcR* and the inducible TPS‐overexpressor line (*iTPS*) were grown from germination in continuous light and temperature (12‐hr/12‐hr light/dark cycle, 160 μmol m^−2^ s^−1^ PFD, 21°C). Three weeks after germination, plants were induced by spraying with 2% (v/v) ethanol (indicated by black arrow) and then darkened 2 hr later. Samples were harvested at 4‐hr intervals during the 24 hr prior to darkening and the first 12 hrs after darkening for measurements of: (a) Tre6P, (b) starch, and (c) sucrose. Values are means ± *SD* (*n* = 4). Significant differences between *AlcR* and *iTPS*, using Student's *t* test, are indicated by asterisks: *p < 0.05, **p < 0.01, and ***p < 0.001. Expanded plots for starch levels in the first 8 hr after darkening and regressions to estimate the rate of starch mobilization are provided in Supporting Information Figure S6. Time series for clock gene transcripts from the same experiment are shown in Supporting Information Figure S5

*AlcR* and *iTPS* had similar and nearly constant levels of starch, sucrose, and Tre6P in continuous light (Figure 7, original data provided in Supporting Information Table S5). It is noteworthy that the leaf starch content in continuous light (about 30 μmol [Glc] g^−1^ FW) was rather low compared to that found in wild‐type, *AlcR,* or *iTPS* in a light‐dark cycle (60–130 μmol [Glc] g^−1^ FW). After darkening, Tre6P declined in *AlcR* controls but remained high in induced *iTPS* plants (average levels in the first 12 hr after darkening were 0.09 ± 0.03 and 0.22 ± 0.07 μmol [Glc] g^−1^ FW, respectively, Figure 7a). Starch mobilization was faster in *AlcR* than *iTPS* (Figure 7b). At 8 hr after darkening, *AlcR* and *iTPS* had remobilized 77% and 33% of their starch, respectively. After this time, the rate of mobilization fell off in the *AlcR* plants. Estimation from the linear regression analysis in the linear phase during the first 8 hr in darkness (Supporting Information Figure S6) revealed a rate of starch mobilization of about 2.1 μmol [Glc] g^−1^ FW hr^−1^ in *AlcR* and 1.14 μmol [Glc] g^−1^ FW hr^−1^ in *iTPS*. We conclude that Tre6P can inhibit starch mobilization in the absence of a synchronized and entrained clock.

### 3.6 Testing whether the clock overrides inhibition by Tre6P

As already mentioned, starch content typically decreases proportionally with the decrease in time to dawn, resulting in a linear depletion of starch and exhaustion of starch at a time around dawn. We reasoned that when Tre6P is inhibiting starch mobilization, the starch content at any given time would be higher than it would if there was no inhibition by Tre6P. Within the context of the molecular division model, this would represent a higher value for ‘*S*’, and lead to a higher value for ‘*R*’ (i.e., a higher rate of mobilization) that would partly compensate for the inhibition by Tre6P. We modeled this interaction, using a numerical Tre6P input that would lead to 0%, 30%, or 60% inhibition of the initial rate of starch mobilization (Supporting Information Figure S7). These values were chosen to span the inhibition seen in the experiments with *sweet11;12* (Figures 1, 2, 3) and the *iTPS* line (Figures 4 and 5) as well as in Figure 7. The predicted inhibition was slightly weaker when the action of Tre6P was modified by a downstream interaction with a clock output (Supporting Information Figure S7B) than in a scenario in which the rate of mobilization was independent of the clock and starch content (Supporting Information Figure S7A). However, the effect was too small to explain why Tre6P often has little or no effect on the absolute rate of starch mobilization (see Figures 1c, 3a–d, 4e, 5e).

## 4 DISCUSSION

As outlined in the Introduction, in C‐limiting conditions, the clock sets the rate of starch mobilization such that starch reserves are almost exhausted at dawn (Scialdone and Howard, 2015; Smith and Stitt 2007; Stitt & Zeeman, 2012), while in C‐replete conditions, starch reserves are not fully exhausted at dawn. Experiments with *iTPS* lines in Martins et al. (2013) pointed to a role for the sucrose signal metabolite Tre6P in the feedback regulation of starch mobilization. Our results confirm and extend the idea that Tre6P regulates starch mobilization in C‐replete conditions. First, impaired sucrose export in *sweet11;12* results in accumulation of sucrose, higher levels of Tre6P, and a restriction of starch mobilization. Second, the relation between Tre6P and starch mobilization in *sweet11;12* is largely recapitulated in *iTPS* lines, even though sucrose and other C‐metabolites rose in the former and decreased in the latter treatment. This points to a dominant role for Tre6P in the feedback regulation of starch mobilization. Third, Tre6P restricts mobilization under a wide range of conditions. However, maybe surprisingly, there is no clear relationship between the level of Tre6P and the absolute rate of starch mobilization. Rather, Tre6P depresses the rate of mobilization below that required to exhaust starch at dawn. As will be discussed, this finding implies that Tre6P interacts closely with the clock to set the rate of starch mobilization.

### 4.1 Elevated Tre6P consistently leads to incomplete exhaustion of starch at dawn but has no consistent impact on the absolute rate of starch mobilization

Our experiments employed two complementary strategies to elevate Tre6P; *sweet11;12* mutants with constitutively higher levels of sucrose and reducing sugars, or transient induction of a bacterial *TPS*, in which case Tre6P levels increased while sucrose and reducing sugars levels fell (see also Martins et al., 2013). We investigated starch mobilization after short‐term changes in the light regime to generate high or low starch levels at dusk. Irrespective of the time frame over which Tre6P levels were elevated and whether the elevation was associated with higher or lower levels of sugars, elevated Tre6P always led to a restriction of starch breakdown, in particular, incomplete mobilization of starch leaving a starch excess at dawn. Crucially, there was a robust negative relationship between the Tre6P level and *R*_r_, the rate of starch mobilization expressed relative to the rate required to exhaust starch at dawn (Figure 6b).

In contrast, there was no consistent relation between Tre6P levels and *R*_a_, the absolute rate of starch mobilization (Figure 6a). In experiments with the *sweet11;12* mutant, elevated Tre6P led to either a minor decrease or even a minor increase in the absolute rate of starch mobilization (Figures 2, 3, 4, Supporting Information Figure S3). In experiments with *iTPS* lines, elevated Tre6P did lead to a decrease in the rate of starch mobilization, but the inhibition was small when they entered the night with low levels of starch (Figure 4). Overall, elevated Tre6P decreased *R*_a_ when plants with elevated Tre6P and control plants entered the night with similar and high levels of starch, but it had only a small effect when they entered the night with similar but low levels of starch, and little or no effect when the plants with elevated Tre6P entered the night with higher levels of starch than control plants (see below for more discussion).

We conclude that Tre6P plays a major role in the feedback regulation of starch mobilization. In particular, elevated Tre6P prevents complete mobilization of starch. The similar relationship between Tre6P levels and *R*_r_ across genotypes underlines the importance of Tre6P in the feedback regulation of starch mobilization. Importantly, elevated Tre6P restricts starch mobilization, irrespective of whether sugars are rising or falling, implying that other C‐signals play only a small role in the feedback regulation of starch mobilization.

### 4.2 Masked relationship between the amount of starch and the rate of starch mobilization

Many earlier studies showed that the rate of starch mobilization in Arabidopsis is fairly constant during the night (i.e., starch content decreases in a linear manner; Flis et al., 2016; Gibon et al., 2004; Graf et al., 2010; Mengin et al., 2017; Scialdone et al., 2013; Sulpice et al., 2014). This was also seen in our study. On the face of it, these observations imply that the rate of starch mobilization is independent of starch content until the latter falls to rather low values.

However, several aspects of our data indicate that a higher starch content does favor a higher absolute rate of starch mobilization. First, in wild‐type plants, higher dusk starch levels typically led to a higher absolute rate of starch mobilization (see e.g., Figures 2c, e and 4c, e, see also Mengin et al., 2017; Scialdone et al., 2013; Pilkington et al., 2015). Second, as already mentioned, the only conditions in which elevated Tre6P led to a large decrease in the absolute rate of starch mobilization (*R*_a_) were when starch levels at the start of the night were high and similar to those in the control (see Figures 6b and 7, Supporting Information Figure S6; also Martins et al., 2013). Third, incomplete mobilization of starch due to feedback inhibition by Tre6P in one night will leave a residue of non‐mobilized starch at dawn (see Figure 4a, B) and, if the rate of starch accumulation during the next day remains unaltered, a higher starch content at the following dusk. This cycle will be repeated until a steady state is reached with a similar absolute rate of starch mobilization (*R*_a_) in the Tre6P‐inhibited case and the control. This new balance can be explained if the inhibition by Tre6P is offset by a higher basal rate of starch mobilization due to the higher starch content. This pattern is seen in the *sweet11;12* mutant in growth conditions (Figure 1d; Supporting Information Figure S4A). It was also seen after overexpressing an activated form of ADPGlc pyrophosphorylase to increase starch accumulation in the light (Hädrich et al., 2012). This response has been modeled more generally for scenarios in which starch mobilization is restricted by factors like decreased starch mobilization capacity or defects in circadian regulation (Seaton et al., 2015).

At first sight, dependence of the rate of mobilization on starch content (‘*S*’) appears to be incompatible with the observed linear decline in starch content during the night (see above). However, this inconsistency can be resolved if a further factor promotes starch mobilization as the 24‐hr cycle progresses, that is, as the remaining time to dawn decreases. One simple and plausible factor is the parameter ‘*T*’ as defined in the arithmetic division model (Scialdone et al., 2013).

### 4.3 Interaction between Tre6P and circadian regulation of starch mobilization

**Figure 8:**
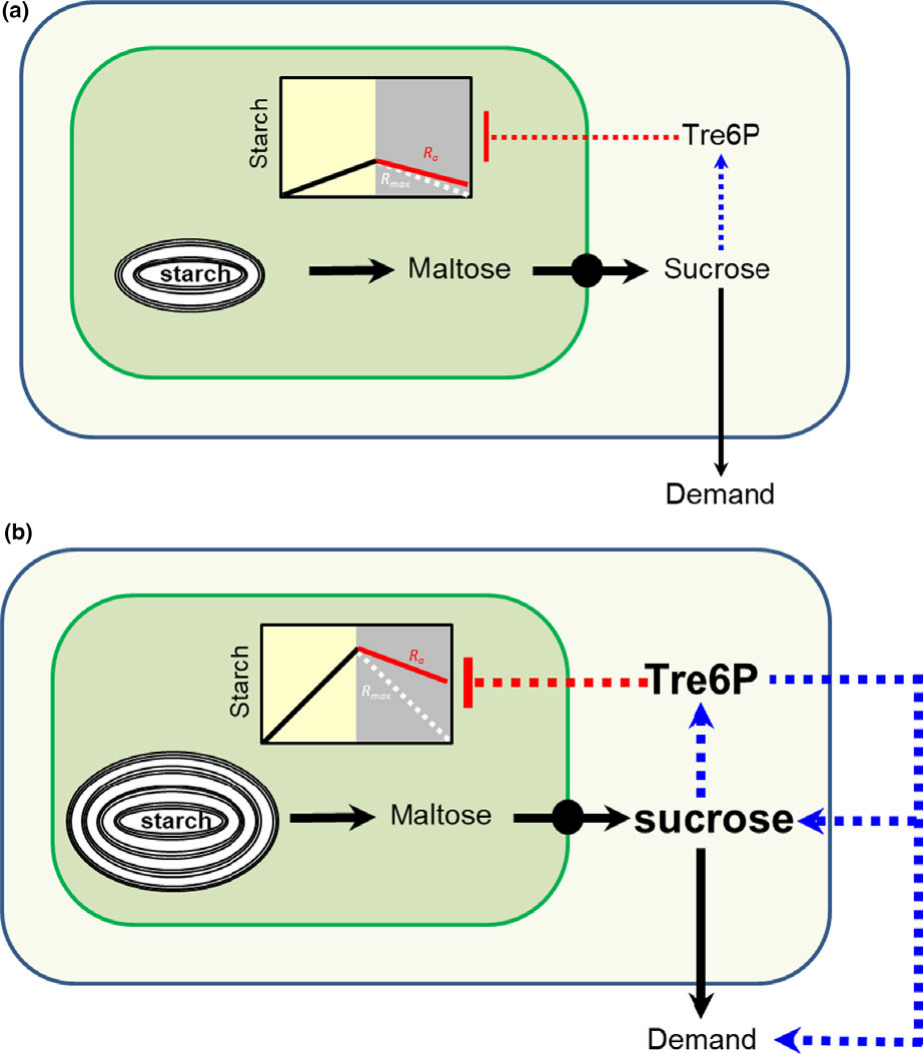
Schematic model to illustrate the inhibitory effect of Tre6P on night‐time starch mobilization in C‐replete and C‐limiting scenarios. In conditions where a low amount of starch is available at dusk, such as in short photoperiod or low irradiance, the clock enforces a low rate of starch mobilization during the night, leading to low levels of sucrose and Tre6P. Tre6P only slightly depresses the relative rate of starch mobilization (*R*_r_) below that permitted by the clock (*R*_max_), and this small depression is further attenuated by the compensatory increase modeled in Supporting Information Figure S7. In conditions where ample starch is available at dusk and starch mobilization supplies more C than can be consumed for metabolism, maintenance and growth, sucrose will increase. The resulting increase in Tre6P will restrict starch mobilization leading to incomplete utilization of starch. High Tre6P (and other C‐signals derived from high sucrose and other sugars) will also act to increase the rate of C utilization. The extent to which starch mobilization is slowed down will depend on the balance between negative feedback of starch mobilization by Tre6P and feedforward signaling by Tre6P and other C‐signals to increase C utilization

In terms of the arithmetic division model, there are several possible explanations for the negative action of Tre6P on starch mobilization: (a) *Tre6P and the clock act independently*. This seems unlikely because the weak and inconsistent impact of Tre6P on the absolute rate of starch mobilization could not be mimicked in a simple model investigating how an independent inhibition by elevated Tre6P would impact on the rate of starch mobilization predicted by the arithmetic division model (Supporting Information Figure S6). This hypothesis also does not explain why elevated Tre6P in iTPS lines led to only a weak and non‐significant decrease in the absolute rate of starch degradation when plants entered the night with low and similar levels of starch to those in control *AlcR* lines (Figures 4c and 5e) (b) *The clock overrides the inhibition by Tre6P*. This seems unlikely, because elevated Tre6P consistently led to an inhibition of starch mobilization (or at least incomplete exhaustion of starch) even under conditions in which dusk starch content was low and mobilization was already slow in the control and, by implication, strongly restricted by the clock. (c) *Tre6P overrides the clock*, that is, modifies ‘*R*’. This also seems unlikely, because the largest inhibition of the absolute rate was seen in a treatment (Figure 6b) in which dusk starch content was high in the control plants, and starch was only partly exhausted at dawn implying that regulation by the clock was rather relaxed. Further, Tre6P inhibited starch mobilization when plants were darkened after 21 days in continuous light, when their transcriptional clock was desynchronized and non‐entrained (Figure 8). (d) *Tre6P interacts with the clock output ‘T’*. This explanation appears unlikely, because Tre6P inhibited starch mobilization in plants with a desynchronized clock. However, it cannot be totally excluded that Tre6P acts on a default value for ‘*T*’. An interaction with a clock output is also a possible explanation in terms of models in which metabolic feedback alters clock period and delays the time at which dawn is anticipated (Seki et al., 2017). However, a short‐term increase in Tre6P at the beginning of the night slows down starch mobilization early in the same night (Figure 6a, b, see also Martins et al., 2013). This rather fast response cannot be easily understood in terms of changes that occur due to C depletion at the following dawn.

The most plausible hypothesis may be that Tre6P interacts with the measure of starch content, ‘*S*’. This provides a simple explanation for the consistent relationship between the level of Tre6P and the proportion of starch that is predicted to be mobilized during the night (*R*_r_, Figure 6b). It also provides a simple explanation why elevated Tre6P only depresses the absolute rate of starch mobilization (*R*_a_) when dusk starch levels are the same in the control and treatments with elevated Tre6P. Furthermore, it is consistent with the finding that Tre6P inhibits starch mobilization in plants with a desynchronized and non‐entrained clock, without requiring additional hypotheses that there is a default measure of time to dawn (‘*T*’) in such conditions. However, it should be noted that within the context of the arithmetic division model, modification of ‘*T*’, and modification of ‘*S*’ will be mathematically similar with respect to the effect on ‘*R*’.

Starch is located in the plastid, but TPS1 lacks a plastid transit sequence and non‐aqueous fractionation indicates that much or even all the Tre6P in the Arabidopsis rosette is located outside the chloroplasts (Martins et al., 2013). The observed inhibition of starch mobilization by Tre6P implies either that some Tre6P does enter the plastid or that Tre6P acts on an extra‐plastidic signaling network that generates signals that enter the plastid to regulate starch mobilization. The reported presence of two Tre6P phosphatase family members in the chloroplast (TPPD and TPPE; Krasensky, Broyart, Rabanal, & Jonak, 2014) means the former scenario deserves further attention, even though there is to date no evidence that Tre6P directly inhibits enzymes involved in starch mobilization (Martins et al., 2013). The core clock operates via transcriptional regulation of gene expression in the nucleus; thus, involvement of the clock in the regulation of starch mobilization implies the movement of a signal from the nucleus or cytosol into the plastid. Changes in Tre6P might be integrated with the clock‐derived signal in either the cytosol or the plastid. The biochemical mechanism that regulates starch mobilization still needs to be elucidated but appears to involve changes in the cycle of starch phosphorylation and dephosphorylation at the surface of the starch granule, irrespective of whether mobilization is responding to the clock (Scialdone et al., 2013) or Tre6P (Martins et al., 2013).

The interaction between the clock and Tre6P in regulating starch mobilization is summarized in Figure 8. In conditions where dusk starch levels are low and the rate of starch mobilization is restricted by the clock, sucrose and Tre6P levels are low at night. This occurs in plants growing in short photoperiods or low irradiance (Mengin et al., 2017; Sulpice et al., 2014) or after a sudden early dusk (Martins et al., 2013). Tre6P will only slightly depress the rate of starch mobilization below that permitted by the clock, and this small depression will be attenuated by the compensation modeled in Supporting Information Figure S7. In conditions where ample starch is available at dusk, starch is rapidly mobilized leading to high levels of sugars and thus Tre6P, which restricts mobilization below the rate that would be permitted by the clock. This occurs in wild‐type plants after treatments to increase the starch content at dusk (see Figures 2a–c, f and 4a–c, f; see also Mengin et al., 2017; Pilkington et al., 2015; Sulpice et al., 2014) and when induced *iTPS* lines enter the night with high starch content (Figure 6). In this interaction, circadian regulation acts to maximize starch mobilization in each 24 hr cycle, while Tre6P restricts starch mobilization when it is supplying more C than can be consumed for metabolism. Importantly, Tre6P not only restricts starch mobilization and sucrose production in leaves. Tre6P also regulates sucrose consumption, including in growing sink organs (Figueroa and Lunn, 2016). For example, Tre6P stimulates utilization of C for amino acid synthesis (Figueroa et al., 2017). Tre6P also stimulates C utilization in the long term by promoting shoot branching and flowering (Fichtner et al., 2017; Wahl et al., 2013). The relative strengths of the inhibitory action of Tre6P on starch mobilization and the positive action of Tre6P and other C‐signals on C utilization will determine to what extent allocation of C to growth is maximized and accumulation of foliar starch is minimized during the day in C‐replete conditions. This balance may depend on the physiological state of the plant, the environment and, likely, the genetic background.

## AUTHOR CONTRIBUTIONS

LdA: Performed experiments and measurements, data analysis, contributed to writing the manuscript; PKP: Performed experiments and measurements, data analysis; TAM: Data analysis; RF: Performed measurements; JEL: Designed experiments, contributed to writing the manuscript; MS: Designed experiments, contributed to writing the manuscript.
