# Supplementary material for: Feedback regulation by trehalose 6‐phosphate slows down starch mobilization below the rate that would exhaust starch reserves at dawn in Arabidopsis leaves
Source: Plant Direct. 2018 Aug 13;2(8):e00078. doi: 10.1002/pld3.78 (PMC6508811; doi:10.1002/pld3.78)
Supplement: Supplementary file 1 [file PLD3-2-e00078-s001.pdf]

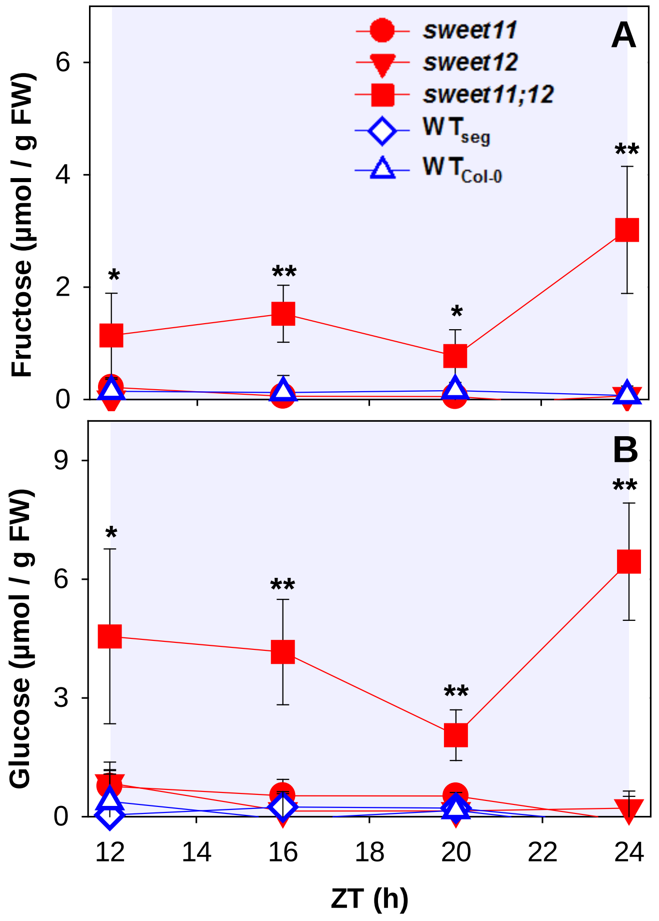

Supplemental Figure S1. Levels of glucose and fructose in the Arabidopsis *sweet* mutants; additional data to the experiment shown in Fig. 1. Rosettes from *sweet11* and *sweet12* mutants, *sweet11;12* double mutant, wild-type segregant (WT_{seg}) and wild type Col-0 (WT_{Col-0}) plants were harvested at various time points during the night. (A) Fructose, (B) Glucose . Values shown are mean ± SD (n = 3). Significant differences between *sweet11;12* and WT_{Col-0} samples , using Student's t-test, are indicated by asterisks *(P < 0.05),** (P < 0.01) and *** (P < 0.001).

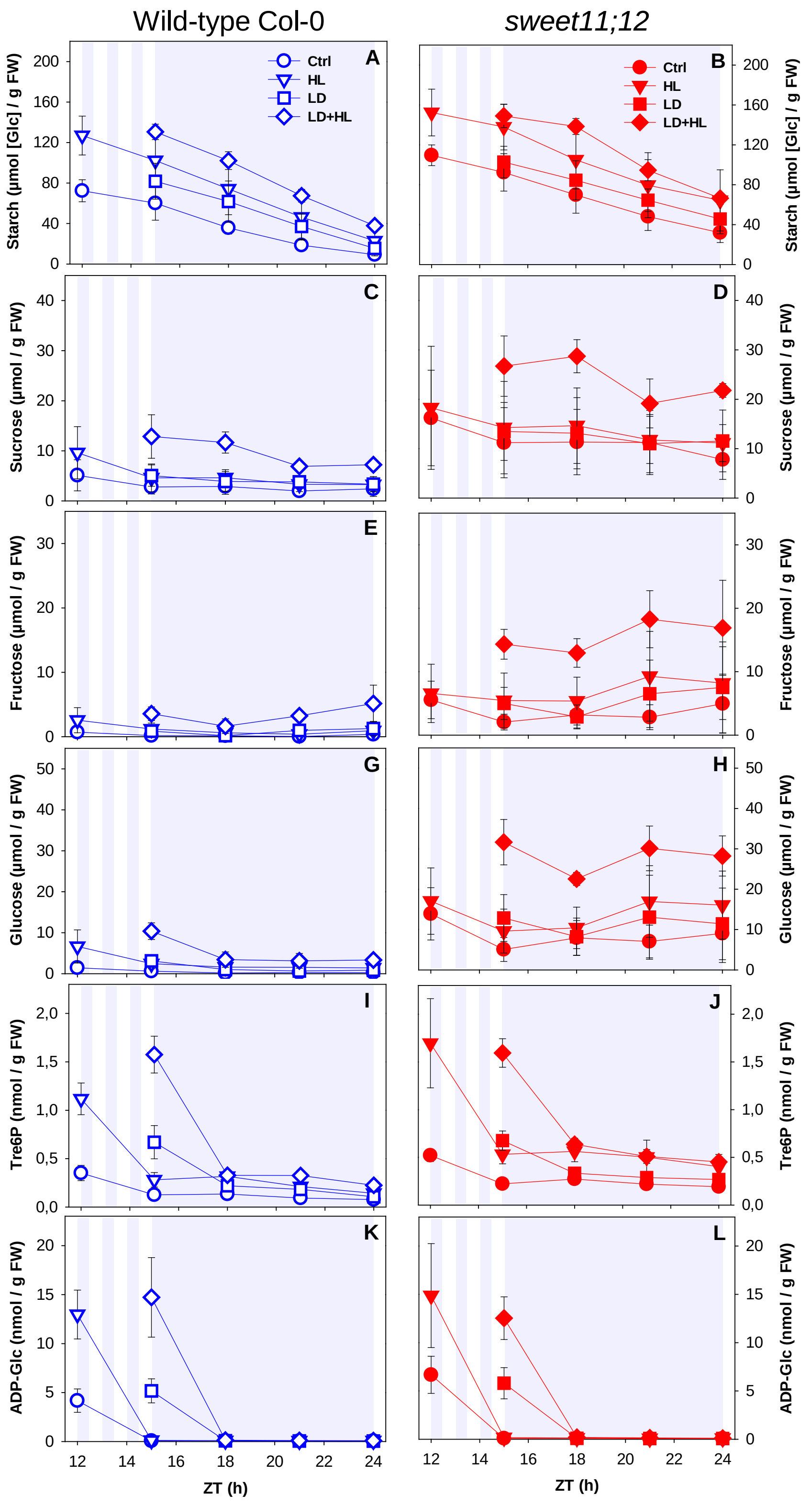

Supplemental Figure S2. Effect of impaired phloem loading in the *sweet11;12* double mutant on night-time metabolite levels. Wild-type Col-0 plants (left column) and *sweet11;12* double mutants (right column) were grown under standard conditions (Ctrl – 12-h(12-h light/dark, 21°C and 160 µmol m^{-2} s^{-1} PFD). Three weeks after germination, batches of plants were left in standard conditions (Ctrl) or were shifted for a single day either to a long-day (LD, 15-h photoperiod, 21°C, 160 µmol m^{-2} s^{-1} PFD), or to high-light (HL, 12-h photoperiod, 21°C, 320 µmol m^{-2} s^{-1} PFD), or a combined long-day and high-light (LD+HL). Samples were collected at the end of the light period (ED) after the shift, and throughout the following dark period at 3-h intervals, up to the end of the night (EN) for metabolite analysis. Values are means ±SD (n=10) taken from two independent experiments. ZT, zeitgeber time (hours after dawn). The hatched area denotes when plants were in darkness in the 12-h light/12-h dark and still in light in the 15-h light/9-h dark treatments. The original data are provided in Suppl. Table S2

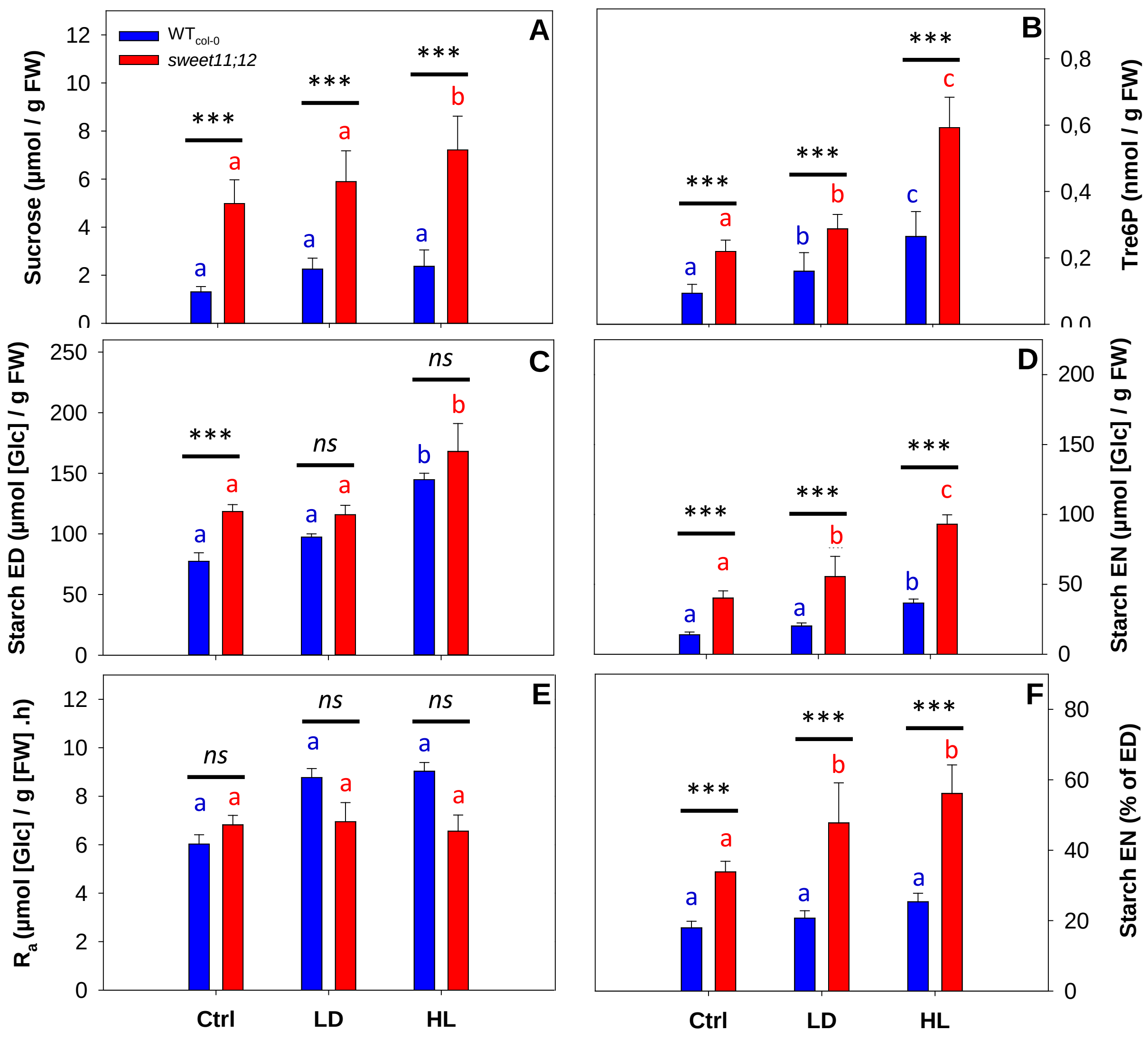

Supplemental Figure S3. Night-time metabolite levels in Col-0 and the *sweet11;12* double mutant exposed in the previous light period to a long day (LD) or high light (HL); the experiment was performed as in Fig. 2, but omitted the combined LD+HL treatment. Wild-type Col-0 plants (WT) and *sweet11;12* double mutants were grown under standard condition (Ctrl – 12-h/12-h light/dark, 21°C and 160 µmol m^{-2} s^{-1} PFD). Three weeks after germination, batches of plants were shifted for a single day either to a long-day (LD, 15-h photoperiod, 21°C, 160 µmol m^{-2} s^{-1} PFD) or high-light condition (HL, 12-h photoperiod, 21°C, 320 µmol m^{-2} s^{-1} PFD) . Metabolites were quantified at the end of the day (ED) after the shift, and throughout the dark period at 3-h intervals up to the end of the night (EN). (A) Night-time sucrose and (B) night-time Tre6P levels are averages from ZT15 to ZT24; (C) starch content at ED; (D) starch content at EN ; (E) Absolute rate of starch mobilization (R_a) estimated as the slope of the linear regression of all starch values ; (F) Starch content at EN expressed as the percentage of starch content at ED. Sucrose and starch were measured enzymatically. Tre6P was determined by LC-MS/MS. Values are means ±SD (n=5). Asterisks indicate statistically significant genotype-dependent differences in a given treatment: * P(< 0.05), ** (P < 0.01) and *** (P < 0.001). Letters indicate significant treatment-dependent differences in a given genotype: blue letters indicate treatment differences within WT$_{Col-0}$ and red letters indicate differences within *sweet11;12* double mutant (one-way ANOVA , Holm-Sidak post hoc pairwise multiple comparison testing, P < 0.01). ZT, zeitgeber time (hours after dawn).

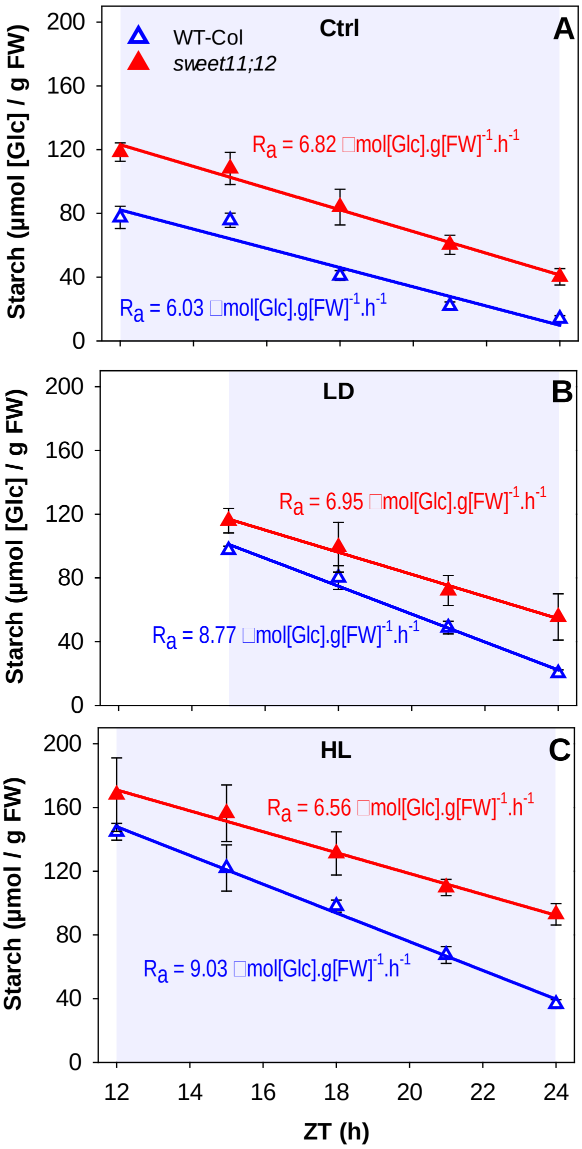

Supplemental Figure S4. Time resolved starch content and estimation of starch mobilization rates in wild-type plants and *sweet11;12* mutants; a further analysis of the data from Suppl. Fig. S3. Wild-type Col-0 plants (WT) and *sweet11;12* double mutants were grown at (A) control standard condition (Ctrl, 12h-L/D cycle, 21°C, 160 µmol m^{-2} s^{-1} PFD). Three weeks after germination, part of the plants were shifted for one single day to (B) long-day condition (LD, 15hL/9hD cycle, 21°C, 160 µmol m^{-2} s^{-1} PFD), or to (C) high-light condition (HL, 12h-L/D cycle, 21°C, 320 µmol m^{-2} s^{-1} PFD). Starch contents were quantified enzymatically at the end of the day (ED) after the shift, and throughout the following dark period at every 3h-interval, up to the end of the night (EN). (A) Lines represent linear regressions calculated for starch contents. Absolute starch mobilization rates (Ra) are indicated in the panels. Values are means ±SD (n=5). ZT, Zeitgeber time (hours after dawn).

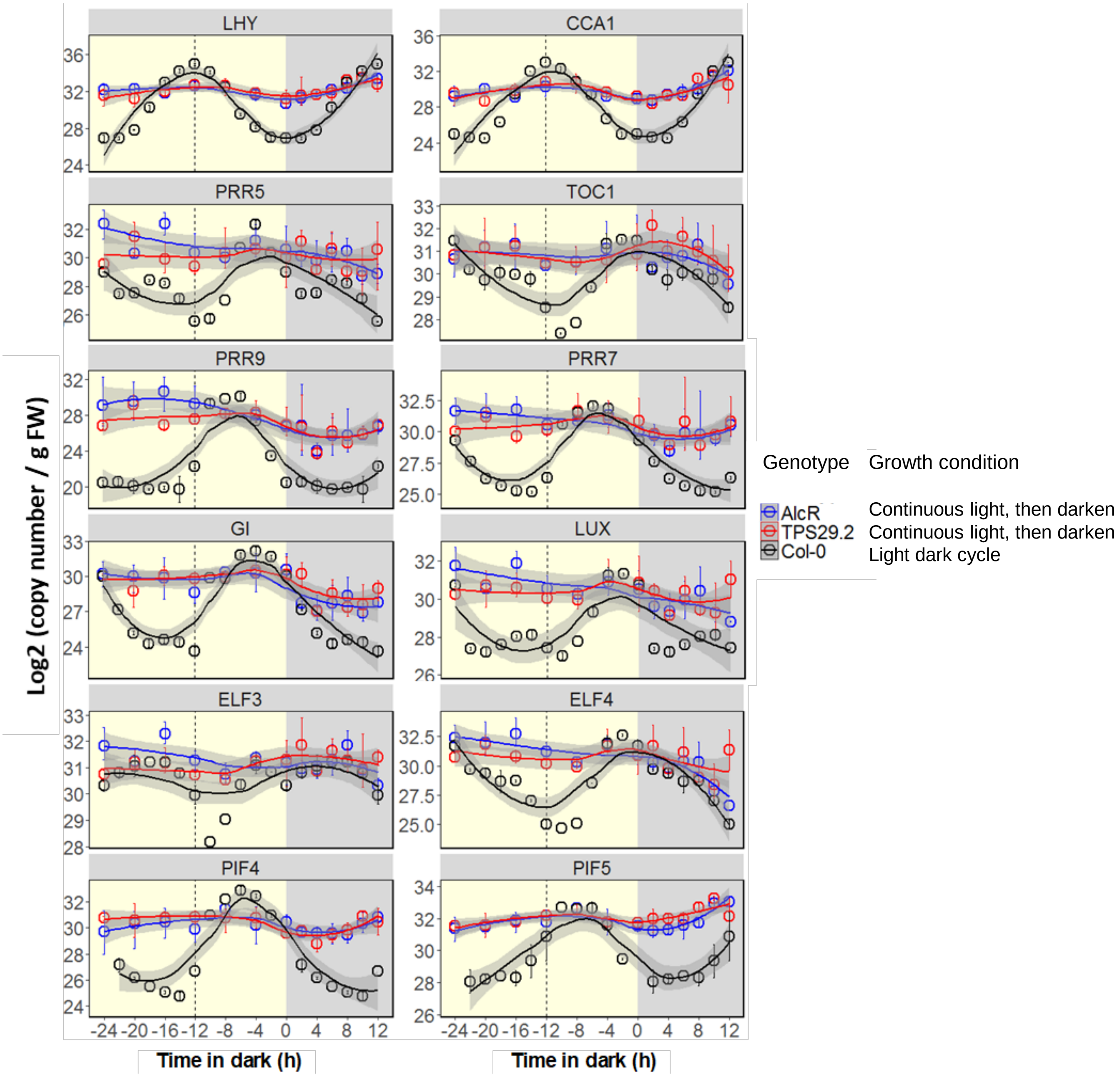

Supplemental Figure S5. Clock gene transcript abundance in Col-0 germinated and grown in continuous light and then transferred to darkness for 12 h compared to Col-0 growing in a 12 h photoperiod; control data to the experiment shown in Fig. 8. The empty-vector control AlcR and the inducible TPS-overexpressor line (*iTPS*) were grown from germination in continuous light and temperature (160 µmol m^{-2} s^{-1} PFD, 21ºC). Three weeks after germination, plants were darkened. Plants were induced 2 h prior to dusk by spraying with 2% (v/v) ethanol. Samples were harvested at 4h-intervals during the 24h prior to darkening and the first 12h hours after darkening. Transcripts were quantified in samples collected at 4h-intervals in the 24h prior to darkening and at 2h-interval in the first 12h hours after darkening. Transcripts were quantified by Real-Time qRT-PCR. Values are means ±SD (n=5). Control wild-type Col-0 data show the response expected in synchronized and entrained plants. The data is taken from Flis *et al.* (2015, 2016); the plants were grown in a 12 h photoperiod (about 150 µmol m^{-2} s^{-1} PFD, 21°C) for 21 days and were then harvested at 2-h intervals through a 24 h light-dark cycle (data is the mean of 2 biological replicates). The data sets are aligned on dusk, i.e., ZT0 in the light-dark data series corresponds to 12 h before darkening in the continuous light treatment (the light-dark cycle data is concatenated). In all cases, transcript abundance is shown as number of copies per grams of fresh weight, on a log2 scale.

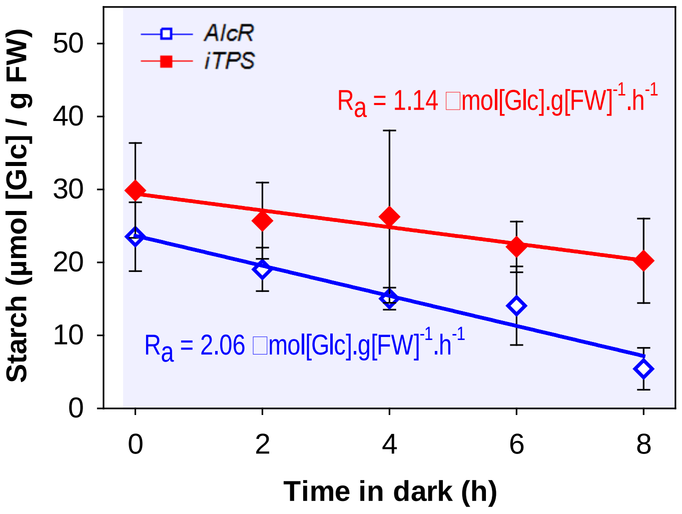

Supplemental Figure S6. Effect of induced TPS overexpression on the rate of starch mobilization when plants germinated and grown in continuous light are suddenly transferred to darkness; this plot analyses the data in Fig. 8, plotting starch content immediately before darkening and during the first 8 h after darkening with a higher time resolution, and using regression to calculate the rate of starch mobilization. For experimental details see the legends of Fig. 8 and Suppl. Fig. S5. Values are means ±SD (n=4). Lines represent linear regressions calculated for starch contents; the absolute starch mobilization rates (R_a) are indicated in the panels.

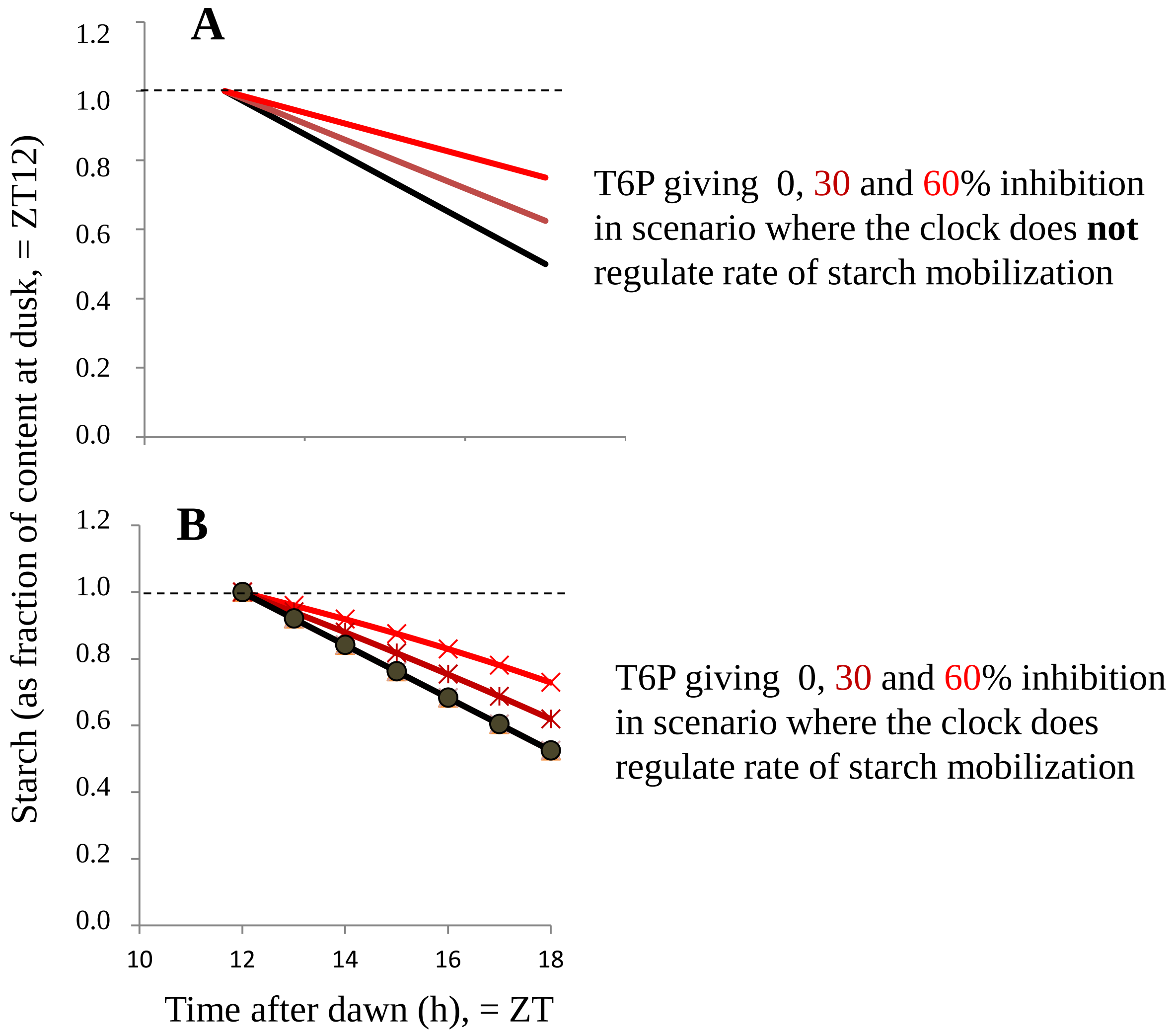

Supplemental Figure S7. Model of the outcome when the inhibitory action of Tre6P occurs downstream of, and modifies, the rate of starch mobilization set by the clock. The upper panel (A) shows a control simulation in which the rate of starch mobilization is reduced by 30 and 60% in a time-independent manner. The lower panel (B) shows a downstream interaction with a clock output that sets the rate of starch mobilization. Briefly, the rate of mobilization, R, is set according to the molecular division model as R = (S/T) where S is the amount of starch at that time and T is the time remaining until dawn. In the absence of any further factors, the set mobilization rate will decrease S proportionately with the decrease in T resulting in a constant rate of mobilization that exhausts starch at dawn. If a further factor like Tre6P depresses the rate of mobilization below R this will, over a time interval, result in a higher level of starch than would otherwise be the case (i.e. S does not decrease proportionally to T). This increase in S relative to T will lead to a higher value of R, partly compensating for the inhibition by Tre6P. Black, brown and red denote models with zero, 30% and 60% inhibition by Tre6P.

The model is explained in more detail, with equations, in Suppl. Table S6.
