# Supplementary material for: Feedback regulation by trehalose 6‐phosphate slows down starch mobilization below the rate that would exhaust starch reserves at dawn in Arabidopsis leaves
Source: Plant Direct. 2018 Aug 13;2(8):e00078. doi: 10.1002/pld3.78 (PMC6508811; doi:10.1002/pld3.78)
Supplement: Supplementary file 8 [file PLD3-2-e00078-s008.pdf]

**Editors comments**	
Reviewer #2 has two major comments about the lack of statistical tests undermining the stated conclusions, suggesting that the review criteria "results support the conclusions" has not been full met. These points must be addressed in your response.	We did not include these analyses in the original version because them main aim of the manuscript is to compare, in a given condition, the responses in between genotypes with a differing level of Tre6P. We were worried that too much discussion of differences between treatments in a given genotype would distract from this main aim We have nevertheless, in response to the request of the reviewer, added all the additional statistics analyses. The way we present them is slightly different from that requested by the reviewer because we do want to focus the readers attention on differences between genotypes in a given condition. We have modified the text where necessary. This has not affected any of our conclusions
The reviewers also have many other critiques that could improve the manuscript but aren't required to be addressed	We have followed the majority of these suggestions (see below for details)

**Reviewer 1**	
be clear whether the terms "starch breakdown". "starch degradation", "starch mobilization" and "starch turnover" refer to the same thing. If yes, I suggest to use for term for the sake of consistency. If different, explain the terms clearly.	We now use mobilization consistently rather than degradation.<br><br>Turnover is different: this term refers to the diel accumulation and mobilization
2 Introduction is too lengthy and could be significantly shortened.<br><br>At the same time, better clarity for certain points should be provided:<br><br>the time to bring our Tre6P is quite late;<br><br>the last sentence of the first paragraph in Introduction and the first sentence of the last paragraph in Introduction can be revised for clarity;<br><br>some introduction to sweet mutant is necessary	We have slightly shorted the introduction be removing discussion of starch degradation in the light<br><br>We have redrafted the end of the first paragraph to introduce Tre6P<br><br>An introduction to the sweet mutants and why we choose this as the tool to decrease phloem export is added<br><br>This allows us to simplify the start of the final paragraph

**Reviewer 2**	
Major comments:	
This manuscript was not carefully proofed prior to submission. Examples of typographical errors are listed below:<br>Line 216. The last sentence misses a parenthesis.	Sorry, corrected

Line 260-263. Why is there a question mark in parentheses at the end of this sentence?<br>Line 266. Fig 1F does not exist.<br>Line 269. The last sentence of this paragraph misses a period.<br>Line 319. Fig. 34 does not exist. "Fig. 4 shows the average Tre6P (Fig. 4) and sucrose (Fig. 34) levels" should be "Fig. 4 shows the average Tre6P (Fig. 4A) and sucrose (Fig. 4B) levels".<br>Line 323. "starch (Fig. 6A-C)" should be "starch (Fig. 5E-F).<br>Line 341. The last sentence of this paragraph misses a period.<br>Line 601. Typo. "were" should be "where".<br>Figure 3 and 5; Supplemental Figure S4 and S6. micromol did not show up correctly in these four figures.<br>Figure 4. Panel 4A was labeled as Tre6P in the figure but stated as sucrose in the figure legend. Panel 4B was labeled as sucrose in the figure but stated as Tre6P in the figure legend.<br>Figure 7 caption. "FigFigure 7" should be "Figure 7".<br>Supplemental Figure S5 legend: Should "quantified at in samples" be "quantified in samples"?. Need a period after "data is concatenated)".	Sorry, removed<br><br>Corrected to 2F<br>Corrected<br><br>Sorry, corrected<br><br><br><br>Corrected<br><br>Corrected<br><br>Corrected<br><br><br><br>Corrected<br><br><br><br>Corrected;<br><br>Corrected;
Line 223-227. The authors did not perform relevant statistical analysis to compare metabolite levels across four different treatments. Therefore, it does not seem appropriate to state that "In wild-type plants starch content at dusk (Fig. 2C) was unaltered in LD and rose in HL and LH+hl, compared to Ctrl". The authors are strongly recommended to perform pairwise comparison of the means (e.g., pairwise t-test or pairwise Tukey's test) among all eight combinations of genotypes and treatments and use letters to indicate the presence and absence of significant difference in Figure 2. For example, values not connected by the same letter are significantly different. This would allow comparison across four different treatments, not just between two genotypes.	WE have added these tests using<br>one-way ANOVA , with Holm-Sidak post hoc pairwise multiple comparison testing,<br><br>The results ate shown to facilitate comparison for genotype-comparisons in a given condition (asterixes in plot, as in previous version) and letters to allow between-condition comparison in a given genotype.<br><br>We chose this display to allow the reader to focus on the between-genotype comparison in a given conditions which is the main aim of the experiment. We also chose this display to avoid comparisons across-genotypes-in-different-conditions, which is not the aim of the experiment and is in this case biologically meaningless.<br><br>The text has been adjusted by specifying significances (see marked up text on lines 218-223). This has not altered the conclusions.
Line 227-228. Relevant statistical analysis (e.g., pairwise comparison of the means) is need to support this statement	This has been done (see last point)
. Some discussion about the interactions between the clock and Tre6P in regulating starch mobilization is rather speculative	We have made it clear that we are discussing hypotheses and also try to make it clear which two hypotheses remain

Figure 2, Figure 4, and Supplemental Figure S3. The authors are strongly recommended to perform pairwise comparison of the means among all the combinations of genotypes and treatments and use letters to indicate the presence and absence of significant difference. This will allow comparisons between different treatments, not just between different genotypes.	This has been done for all three figures / suppl figures (see above)<br><br>The text has been edited to specify the results of the additional tests (se marked up text in the paragraphs at lines 212-213, 218-223, 268-286 and lines 332-347. This has not altered any of our conclusions
Minor comments	
Line 251-252. The authors may consider deleting one of the two "dusk" in this sentence. More importantly, the dust starch content in wild-type plants under LD does not seem to be higher than that under standard conditions (Ctrl) (Figure 2C). The authors may want to revise this sentence accordingly	The reviewer is correct. We have rephrased this: 'Comparing across treatments, wild-type plants mobilized their starch slightly faster after the LD and considerable faster after the HL and LD+HL treatments than in the control (5.16, 6.14, 8.59 and 9.71 µmol[Glc] g^{-1}FW h^{-1} in Crtl, LD, HL and LD+HL, respectively). The increase in HL and LF+HL reflects an increased dusk starch content.'
Line 273-275. The authors stated that "Compared to the experiment of Figs 2-3, wild type plants contained slightly more starch at ED (Suppl. Fig. S3C) and considerably more starch at EN (Suppl. Fig. S3D)." But I do not see this. Am I missing something?	We have checked this and our statement is correct – it is necessary to ignore the LD+HL treatment in making the comparisons. This is not an important point but we felt I important to state that there was this difference and offer an explanation, as otherwise some readers may also see the issue and wonder why we do not mention it
Line 420-421. The authors may want to mention the decline of sucrose level at the last 8 hours of continuous light (Figure 7C).	This decline is not significant
Supplemental Figure S5. The labels of X-axis in both panels are too far from the panels.	We have looked at the figure again, and do not think the x-axis labels are too far away
Supplemental Figure S7. The authors may consider replacing the commas in numbers on the y-axis with dots. The authors are recommended to change the line for 0% inhibition from black to brown to match with the color codes.	Thank you for seeing this.<br>We have changed the 0% inhibition to black in the caption (we think this is clearer than using an additional brown shade in the panel)
